# Selenium nanoparticles alleviate renal ischemia/reperfusion injury by inhibiting ferritinophagy via the XBP1/NCOA4 pathway

**DOI:** 10.1186/s12964-024-01751-2

**Published:** 2024-07-25

**Authors:** Zhenying Zuo, Mianna Luo, Zhongyu Liu, Ting Liu, Xi Wang, Xiaorong Huang, Shangmei Li, Hongluan Wu, Qingjun Pan, Tianfeng Chen, Lawei Yang, Hua-Feng Liu

**Affiliations:** 1 Guangdong Provincial Key Laboratory of Autophagy and Major Chronic Non-Communicable Diseases, Affiliated Hospital of Guangdong Medical University, Zhanjiang, China; 2 Key Laboratory of Prevention and Management of Chronic Kidney Disease of Zhanjiang City, Institute of Nephrology, Affiliated Hospital of Guangdong Medical University, Zhanjiang, China; 3 Department of Chemistry, Jinan University, Guangzhou, China

**Keywords:** Selenium nanoparticles, X-box binding protein 1, Ferritinophagy, Ferroptosis, Ischemia/reperfusion, Acute kidney injury

## Abstract

Acute kidney injury (AKI) is closely related to lysosomal dysfunction and ferroptosis in renal tubular epithelial cells (TECs), for which effective treatments are urgently needed. Although selenium nanoparticles (SeNPs) have emerged as promising candidates for AKI therapy, their underlying mechanisms have not been fully elucidated. Here, we investigated the effect of SeNPs on hypoxia/reoxygenation (H/R)-induced ferroptosis and lysosomal dysfunction in TECs in vitro and evaluated their efficacy in a murine model of ischemia/reperfusion (I/R)-AKI. We observed that H/R-induced ferroptosis was accompanied by lysosomal Fe^2+^ accumulation and dysfunction in TECs, which was ameliorated by SeNPs administration. Furthermore, SeNPs protected C57BL/6 mice against I/R-induced inflammation and ferroptosis. Mechanistically, we found that lysosomal Fe^2+^ accumulation and ferroptosis were associated with the excessive activation of NCOA4-mediated ferritinophagy, a process mitigated by SeNPs through the upregulation of X-box binding protein 1 (XBP1). Downregulation of XBP1 promoted ferritinophagy and partially counteracted the protective effects of SeNPs on ferroptosis inhibition in TECs. Overall, our findings revealed a novel role for SeNPs in modulating ferritinophagy, thereby improving lysosomal function and attenuating ferroptosis of TECs in I/R-AKI. These results provide evidence for the potential application of SeNPs as therapeutic agents for the prevention and treatment of AKI.

**Supplementary Information:**

The online version contains supplementary material available at 10.1186/s12964-024-01751-2.

## Introduction

Acute kidney injury (AKI) is a severe clinical syndrome typically characterized by a sudden decrease in glomerular filtration rate (GFR), often evidenced by rapid increases in serum creatinine levels and/or a decrease in urine output, which leads to electrolyte disturbance, metabolic acidosis, and volume overload [1]. The morbidity and mortality of AKI are increasing, and AKI is recognized as an important risk factor for chronic kidney disease (CKD) [2]. Patients with AKI face an increased risk of developing CKD (8.8 fold) and end-stage renal disease (ESRD) (3.1 fold) [3]. Renal ischemia/reperfusion (I/R) injury, resulting from kidney transplantation, cardiac arrest hypotension or shock, is a leading etiological factor of AKI [4, 5]. Therefore, identifying novel therapeutic targets and drugs for preventing and treating I/R-AKI is imperative.

Renal tubular epithelial cells (TECs) are particularly vulnerable to injury, marked by various forms of cell death, and inflammation caused by macrophages and damage-related molecular patterns, which is a critical early event in the development of I/R-AKI [6, 7]. Our previous studies have demonstrated that impaired lysosome function and insufficient lysosome biogenesis significantly contributed to renal tubular injury and loss [8, 9], suggesting that maintaining lysosomal homeostasis could be a promising therapeutic strategy for I/R-AKI.

Ferroptosis, a recently recognized form of programmed cell death driven by iron-dependent lipid peroxidation via the Fenton reaction, plays a pivotal role in the pathophysiology of I/R-AKI [10]. Our study showed that high-iron microenvironment stimulation induced lipid peroxidation in alveolar epithelial cells, exacerbating the progression of acute lung injury to pulmonary fibrosis [11]. Recent findings suggest that lysosomal iron accumulation activates ferroptotic signaling pathways [12]. Growing evidence indicates that ferritinophagy, a well-programmed process of nuclear receptor coactivator 4 (NCOA4)-mediated degradation of ferritin and subsequent release of Fe^2+^ within lysosomes, contributes to the induction of ferroptosis [13, 14]. However, the precise mechanism underlying the role of ferritinophagy in I/R-AKI remains unknown.

Selenium nanoparticles (SeNPs) are high-density selenium particles formed from zero-valent selenium with nanoscale characteristics [15]. Researchers, including ours, have highlighted the diverse biological functions of SeNPs, such as anti-tumor, antioxidation and anti-inflammatory properties [16–18]. Recent studies have confirmed that SeNPs could alleviate I/R-AKI by enhancing the expression of selenoprotein glutathione peroxidase 1 (GPX1) and other mechanisms [19, 20]. However, the impact of SeNPs on lysosomes and ferritinophagy in I/R-AKI remains poorly understood.

In this study, we aimed to elucidate the molecular mechanisms of ferroptosis in TECs in context of I/R-AKI and investigated the cytoprotective effect of SeNPs. Our findings provide novel insights into the role of SeNPs in blocking ferritinophagy via the XBP1/NCOA4 signaling, thereby reducing lysosomal Fe^2+^ accumulation and increasing TEC resistance to ferroptosis. Overall, this study sheds light on the unique renoprotection mechanism of SeNPs and suggests that SeNPs are promising therapeutic agents for the prevention and treatment of I/R-AKI.

## Materials and methods

### Human clinical samples

Renal biopsy specimens from patients with AKI and minimal change disease (MCD) were obtained from the Department of Nephrology, Affiliated Hospital of Guangdong Medical University, with the approval of the Institutional Review Board of the Affiliated Hospital of Guangdong Medical University (No. YS2022259). Patients with AKI were pathologically diagnosed with acute renal tubular necrosis, whereas MCD controls exhibited no detectable tubular lesions verified via renal biopsy. The exclusion criteria included previous CKD, pre-biopsy immunosuppressant use, and AKI secondary to other glomerular diseases (e.g., neolunar glomerulonephritis, vasculitis IgA nephropathy, etc.). Patients provided informed consent for the use of their kidney tissue for medical research. The clinical data of the patients are shown in Table 1.

**Table 1:** Baseline data of renal biopsy in AKI and MCD patients

Indexes	AKI patients (*n* = 20)	MCD patients (*n* = 20)	*P*
Female, n (%)	7 (35.0%)	7 (35.0%)	1.000
Age, median (IQR)	49.5 (41.3–52.0)	47.0 (33.0–54.0)	0.946
Systolic pressure (mmHg), mean (S.D.)	133.6 (17.1)	135.6 (18.9)	0.734
Diastolic pressure (mmHg), mean (S.D.)	81.2 (11.1)	82.3 (10.7)	0.752
Hemoglobin (g/L), mean (S.D.)	127.0 (22.9)	137.8 (18.3)	0.109
Blood urea nitrogen (mmol/L), mean (S.D.)	16.4 (8.9)	4.8 (1.0)	0.000**
Serum creatinine (µmol/L), median (IQR)	334.5 (141.5–547)	62.0 (52.0-70.8)	0.000**
GFR (ml/min/1.73m^2^), median (IQR)	17.2 (9.4–47.1)	108.9 (98.9-127.1)	0.000**
Uric acid (µmol/L), mean (S.D.)	476.5 (174.2)	400.2 (80.4)	0.083
Albumin (g/L), mean (S.D.)	31.7 (9.1)	26.0 (10.2)	0.07
Triglyceride (mmol/L), median (IQR)	2.2 (1.5–2.8)	2.2 (1.6–3.5)	0.903
Cholesterol (mmol/L), mean (S.D.)	5.9 (2.8)	8.6 (3.5)	0.01*

Continuous variables are expressed as mean ± SD if normal distribution, as median (interquartile range) if non-normal distribution. Categorical variables are expressed as counts and percentages. Comparisons are based on Wilcoxon rank-sum test, t-test, or chi-square test

*P < 0.05, ** *P* < 0.001

### Preparation of renal I/R injury model

Male 8-week-old C57BL/6 mice were purchased from Liaoning Changsheng Biotechnology Co., Ltd. (Liaoning, China, Quality certificate No. 210,726,220,100,992,385) and housed under standardized pathogen-free conditions (22–24℃) with a 12-hour light/dark cycle with freely available water and food.

The mice were randomly assigned to the following groups: control (Sham, *n* = 5), SeNPs (*n* = 6), I/R (*n* = 6) and I/R + SeNPs (*n* = 6) groups. Briefly, mice were intravenously injected with or without SeNPs at a dose of 0.04 mg/kg 2 h prior to unilateral I/R. Renal I/R injury was induced by clamping the left renal pedicles for 30 min under anesthesia. The sham-operated mice underwent the same procedure, without clamping of the left renal pedicles. After surgery, the mice had free access to water and food. To evaluate renal function, right nephrectomy was performed 24 h before the mice were sacrificed. Mice were euthanized 3 days post-reperfusion, and the left kidney and blood samples were collected and subjected to detection, including histological evaluation, protein extraction, analysis of serum creatinine (Scr) and blood urea nitrogen (BUN). All animal experiments were carried out with the approval of the Animal Experimental Ethics Committee of the Affiliated Hospital of Guangdong Medical University (AHGDMU-LAC-I(1)-2208-B019). All animal procedures were performed in strict accordance with the National Institutes of Health Guide for the Care and Use of Laboratory Animals.

### Histological assessments

Renal tissue samples were immersed and fixed in 4% paraformaldehyde (Sigma-Aldrich, P6148, USA) for 24 h, dehydrated through an ethanol series, and then paraffin-embedded. Renal tissue slices (3 μm) were stained with hematoxylin and eosin (H&E, Solarbio, G1080, China) for morphological examination under a light microscope ( 400× magnification). Histological scoring was performed by grading tubular necrosis, loss of brush border, cast formation, and tubular dilatation as a percentage of the whole cortical area of the kidney slices as follows: 0, none; 1, < 10%; 2, 11–25%; 3, 26–45%; 4, 46–75%; and 5, > 76% [21–23].

### Measurement of kidney function

The blood of the mice was centrifuged at 3000 g and 4 °C for 10 min, and the supernatant was aspirated to obtain the serum. Serum creatinine (Scr) and blood urea nitrogen (BUN) levels were measured using a creatinine kit (Jiancheng Bio, C011-2-1, China) and a BUN kit (Jiancheng Bio, C013-2, China), respectively, according to the manufacturer’s instructions.

### Cells and cell culture

Human proximal tubular HK-2 cells (ATCC, CRL-2190TM, USA) were maintained in Dulbecco’s modified Eagle’s medium (DMEM, Gibco, C11995500BT, USA) supplemented with 10% fetal bovine serum (FBS) (Gibco, 10,082,147, USA) and 1% penicillin and streptomycin (Gibco, 15140-122, USA). The cells were cultured in a cell incubator (Thermo Scientific, HERAcell 150i, USA) with a humidified 5% CO_2_ atmosphere at 37 °C.

### Lentivirus and infection

Human XBP1 (NC_000022.11)-knockdown lentiviral particles were purchased from Genechem Co., Ltd. (Shanghai, China), and an empty vector was used as a negative control (NC). Infection with XBP1-knockdown (XBP1^KD^) lentivirus and the empty vector was performed according to the manufacturer’s instructions.

### Preparation of H/R model in vitro

To mimic H/R conditions in vitro as reported previously [24], cells were treated with varying concentrations of the hypoxia inducer cobalt (II) chloride hexahydrate (CoCl_2_) (Sigma-Aldrich, C8661, USA) for 24 h, followed by CoCl_2_ removal and reoxygenation for different durations (3, 6, 9, 12, and 24 h). Hypoxia conditions were also created with some modifications, as previously reported [25, 26]. Briefly, hypoxia conditions were induced by incubating the cells in serum-free medium within an anaerobic chamber (ESCO, CCL-050T-8, Singapore) equilibrated with 1% O_2_, 5% CO_2_ and 94% N_2_ at 37 °C for 24 h. Subsequently, the cells were reoxygenated with 2% FBS under normoxia conditions in a humidified atmosphere (95% air/5% CO_2_) at 37 °C for the specified time. The control cells were cultured under normoxia conditions.

### Synthesis and characterization of LNT-SeNPs

LNT-SeNPs used in this study, abbreviated as SeNPs, was designed and synthesized from Tianfeng Chen, PhD (Department of Chemistry, Jinan University, Guangzhou, China). Briefly, vitamin C (40 mM, Sigma-Aldrich, A4403, USA) was added dropwise into an equal volume of a Na_2_SeO_3_ (10 mM, Sigma-Aldrich, S9133, USA) and lentinan (4 mg/ml, Selleck, S5083, USA) mixed solution, and the mixture was kept at 25 °C and stirred for 8 h. The obtained nanoparticles were purified by dialyzing the solution with distilled water for 48 h at room temperature to remove excessive reactants. Coupled Plasma Mass Spectrometry (ICP-MS) was used to determine the concentration of Se in SeNPs. The hydrodynamic size and the surface charge of the synthesized SeNPs were detemined by Zetasizer Nano ZS particle analyzer (Malvern Instruments Limited, UK). Images of SeNPs were photographed by transmission electron microscope (Hitachi, H-7650, Japan).

### Chemical treatment and antibodies

CoCl_2_ and chloroquine (CQ, Sigma-Aldrich, C6628, USA) dissolved in phosphate-buffered saline (PBS, Invitrogen, 10,010,023, USA), deferoxamine (DFO, Sigma-Aldrich, D9533, USA) dissolved in distilled water, ferrostatin-1 (Fer-1, Sigma-Aldrich, SML0583, USA), necrostatin-1 (Nec-1, Sigma-Aldrich, N9037, USA), and Z-VAD-fmk (Selleck, S7023, USA) dissolved in dimethyl sulfoxide (DMSO, Sigma-Aldrich, D2650, USA) were supplemented into the medium at final concentrations as described in each experiment. Cells grown to 60% confluency were pretreated with SeNPs, DFO, Fer-1, Nec-1, Z-VAD-FMK or CQ for 1 h prior to H/R treatment. To simulate various H/R conditions in vitro, cells were exposed to different concentrations of the hypoxia inducer CoCl_2_ for 24 h, followed by CoCl_2_ removal and reoxygenation for various durations.

The antibodies used and their sources are as follows. GPX4 (ab125066), ferritin (ab75973), XBP1 (ab37151), phospho-mixed lineage kinase domain-like protein (p-MLKL, ab196436), 4-hydroxynonenal (4-HNE, ab46545) and lysosome-associated membrane protein 1 (LAMP1, ab25630) were purchased from Abcam Biotechnology in the UK; antibodies against-poly ADP-ribose polymerase (PARP, sc-8007), ARA70/NCOA4 (sc-373,739), ferritin (sc-376,594) and β-actin (sc-47,778) were from Santa Cruz Biotechnology in the USA; and solute carrier family 7 member 11 (SLC7A11, #12,691) and MLKL (#14,993) were purchased from Cell Signaling Technology in USA; anti-HAVCR1/TIM1/kidney injury molecule (KIM-1) (R&D Systems, AF1817, USA); anti-GAPDH (Absin, abs830030, China); F4/80 (Proteintech, 29414-1-AP, China); achaete-scute family BHLH transcription factor 4 (ACSL4, Affinity Biosciences, DF12141, USA); goat anti-rabbit (401,315), goat anti-mouse (401,215) and donkey anti-goat (AP180P) secondary antibodies conjugated to horseradish peroxidase (HRP) were from Sigma-Aldrich in the USA; donkey anti-rabbit (A21206) and donkey anti-mouse (A21203) secondary antibodies conjugated with Alexa Fluor 488 or 594 dye were from Invitrogen in the USA.

### Western blotting analysis

Total protein extracts from renal cortex tissue or HK-2 cells were lysed in radioimmunoprecipitation assay (RIPA) lysis buffer (Beyotime, P0013B, China) containing a protease inhibitor cocktail (Beyotime, ST506, China) and phosphatase inhibitor (Applygen, P1260, China) on ice for 15 min. After centrifugation at 13,200 g for 15 min at 4 °C, the supernatants were collected and quantified using a BCA Protein Assay Kit (Thermo Fisher Scientific, 23,227, USA) according to the manufacturer’s instructions. Equal amounts of cell lysates (10–30 µg) was separated by 10–12% sodium dodecyl sulfate-polyacrylamide gel electrophoresis (SDS-PAGE) and electroblotted onto a polyvinylidene difluoride membrane (Millipore, ISEQ00010, USA). The membranes were blocked with 5% (w/v) bovine serum albumin (BSA, Sigma-Aldrich, V900933, USA) for 1 h at room temperature (RT) and then incubated with a 1:2,000 dilution of primary antibodies overnight at 4 °C, followed by incubation with anti-rabbit, anti-mouse or anti-goat HRP-conjugated secondary antibody for 1 h at RT. Finally, the blots were then visualized by Clarity Western ECL Substrate (Bio-Rad, 1,705,061, USA) on a C500 Imaging System (Azure Biosystems, USA).

### Immunofluorescence

Frozen kidney sections or HK-2 cells cultured on coverslips were fixed with 4% paraformaldehyde in PBS for 10 min at RT and subsequently permeabilized with 0.5% Triton X-100 (Sigma-Aldrich, T8787) in PBS for 10 min. After blocking with 1% BSA and subsequent incubation with primary antibody in a humid chamber for 3 h at RT or overnight at 4℃. The sections or cells were further incubated with Alexa Fluor-conjugated secondary antibodies for 1 h at RT in the dark. 4’,6-diamidino-2-phenylindole (DAPI, Beyotime, C1005, China) was used for nuclear staining. Immunofluorescence imaging was performed using a fluorescence microscope (Olympus, FV3000, Japan).

### Immunohistochemistry

Renal tissue slices (3 μm) were prepared as detailed in section of “**Histological assessments**”. These slices were dewaxed according to the published protocol [27]. Antigen retrieval was performed by heating in boiled antigen retrieval buffer (Beyotime, P0083, China) at 100 °C for 10 min. After blocking, the slices were incubated with primary antibodies for 3 h at RT or overnight at 4℃, followed by incubation with HRP-conjugated secondary antibodies for 1 h at RT and with DAB substrate (ZSGB, ZLI-9017, China) respectively. Hematoxylin was used for nuclear staining. Immunohistochemistry imaging was performed using a light microscope (Olympus, IX81, Japan). Ten images at 400× magnification were randomly chosen for quantifying the immunohistochemical staining intensity using ImageJ software. The area of positive staining was delineated and this was calculated as a percentage of the total cortical area of the kidney tissue.

### Transmission electron microscopy

Ultrastructure analysis of renal proximal tubule cells was conducted using transmission electron microscope (Jeol Ltd., JEM-1400HC, Japan). Fixation, dehydration, embedment and staining of human and mouse renal tissue samples were performed as previously described [28]. Ultrathin sections were examined under a transmission electron microscope.

### Cell viability assay

HK-2 cells were plated onto 96-well plates at seeding densities of 6$\times$10^3^ cells per well. Cells were exposed to appropriate treatments in the presence or absence of H/R at 60% confluency. After treatment, thiazolyl blue tetrazolium bromide (MTT, Sigma-Aldrich, M5655, USA) solution was added to each well (at final concentration of 200 µg/mL). Following a 2-hour incubation at 37℃, the supernatants were carefully removed, and the formazan crystals were dissolved in DMSO (200 µl/well). The absorbance was measured at 570 nm using a microplate spectrophotometer (BioTek, ELX800, USA). Cell viability was expressed as a percentage versus untreated control cells.

### DQ-ovalbumin dequenching assay

DQ-Ovalbumin (Invitrogen, D12053, USA) staining and assay were conducted as previously described [8].

### Lipid ROS detection

HK-2 cells seeded in glass-bottom dishes (Beyotime, FCFC020, China) were pretreated with SeNPs for 1 h before exposure to H/R or normoxia conditions. Subsequently, the cells were washed twice with PBS and incubated with the lipid reactive oxygen species (ROS) probes C11-BODIPY^581/591^ (Invitrogen, D3861, USA) (2 µM in serum-free DMEM) and Hoechst 33,342 (Beyotime, C1027, China) at 37 °C for 30 min. After washing away the unbound probes, the cells were transferred to serum-free DMEM. Direct imaging of lipid ROS in cells loaded with oxidized C11-BODIPY (510 nm) was performed using a fluorescence microscope (Olympus, FV3000, Japan).

#### Mitochondrial membrane potential detection

HK-2 cells were subjected to H/R in the presence or absence of SeNPs. The mitochondrial membrane potential was detected using JC-1 probe (Invitrogen, M34152, USA) under a fluorescence microscope (Olympus, FV3000, Japan). Briefly, the cells were washed twice with PBS and incubated with the JC-1 probes (2 µM in serum-free DMEM) at 37 °C for 30 min. Direct imaging of the cells loaded with JC-1 was performed by fluorescence microscope (Olympus, FV3000, Japan).

### Plasmid transfection and generation of stably transfected cell line

The LAMP1-mGFP plasmid (Addgene, #34,831, USA) was transfected into HK-2 cells using Lipofectamine 3000 (Invitrogen, L3000150, USA) according to the manufacturer’s instructions. Six hours after transfection, the cells were incubated in antibiotic-free culture medium. Cell passaging was performed when the cells reached 90% confluence. After one week, HK-2 cells transfected with the LAMP1-mGFP plasmid were isolated by sterile flow sorting using a flow cytometer (BD, FACSAria II, USA).

### Cellular and lysosomal Fe^2+^ detection

HK-2 cells stably expressing LAMP1-mGFP were plated in glass-bottom dishes or 12-well plates and treated as indicated. After washing with serum-free DMEM three times, the cells in the glass-bottom dishes were switched to 1 ml of serum-free DMEM containing 1 µM of the Fe^2+^ probes FerroOrange (Dojindo, F374, Japan) and Hoechst 33,342 solution. The cells were then incubated for 30 min within a 37℃ incubator equilibrated with 95% air and 5% CO_2_. Fluorescently stained cells were then observed under a fluorescence microscopy. Cells plated in 12-well plates were washed three times with PBS, trypsinized and centrifuged. The cell pellet was suspended, washed three times with PBS, and incubated with 1 µM of FerroOrange in serum-free medium for 15 min at 37℃. The stained cells were detected using a flow cytometer (BD, FACSCantoII, USA) and analyzed using FlowJo software (BD, v10, USA).

### Intracellular localization of SeNPs

The intracellular localization of SeNPs was monitored by using a fluorescence microscope. In brief, HK-2 cells were plated in glass-bottom dishes for 24 h. Cells were then washed three times with PBS and stained with 70 nM of LysoTracker Red (Invitrogen, L7528, USA) and Hoechst 33,342 solution (Beyotime, C1027, China) for 10 min. After washing three times with PBS, the cells were incubated with 2 µM of SeNPs-GFP for various durations (0, 15, 30, 45 and 60 min). After three additional washes with serum-free DMEM, the cells in the glass-bottom dish were switched to 1 ml of serum-free DMEM and the intracellular localization of SeNPs-GFP was examined using a fluorescence microscope (Olympus, FV3000, Japan).

### RNA-seq analysis

RNA-seq analysis was performed at BGI Genomics Co., Ltd. (Shenzhen, China). Total RNA was extracted from HK-2 cells subjected to different treatments, including SeNPs alone, H/R, and SeNPs combined with H/R, and renal cortex samples from I/R-AKI mice with or without SeNPs supplementation. After quality inspection, a certain amount of total RNA samples were denatured at suitable temperature to open their secondary structure, and mRNA was enriched by oligo (dT)-attached magnetic beads, followed by mRNA fragmentation, cDNA synthesis and the end-repair, adding ‘A’ nucleotides to the 3’ ends of the blunt fragments as well as adaptor ligation of the cDNA. PCR amplification was then performed and the products were submitted for cDNA library construction. The prepared libraries were sequenced using high-intensity DNA nanochip techniques and combinatorial probe-anchor synthesis (cPAS). Differential expression analysis was performed using the DESeq R package. Genes with an adjusted *P-*value < 0.05 were considered differentially expressed.

### Single-cell transcriptomic sequencing analysis of the mouse kidney

A mouse model of unilateral renal I/R-AKI was established according to the experimental method outlined in “**Preparation of renal I/R injury model**”. Following blood collection from the right ventricle of the mice, approximately 20 ml of pre-cooled PBS was injected into the left ventricle until both kidneys turned white. Subsequently, the renal capsule was removed, the left kidney was rinsed in pre-cooled PBS before being immersed in 1 ml of fixative solution. These tissue samples were transported to Suzhou Newgogene Biotech Co., Ltd. for single-cell transcriptomic sequencing analysis.

### Statistical analysis

Results are presented as the mean ± standard error from at least three independent experiments. Statistical analyses were performed using GraphPad Prism (version 8.0, GraphPad Software, Inc). One-way analysis of variance with Tukey’s post-hoc test was used to compare parameters among groups. Statistical significance was defined as *p*-value < 0.05.

## Results

### Ferroptosis is induced in TECs of mice after I/R injury and in patients with AKI

**Fig. 1:**
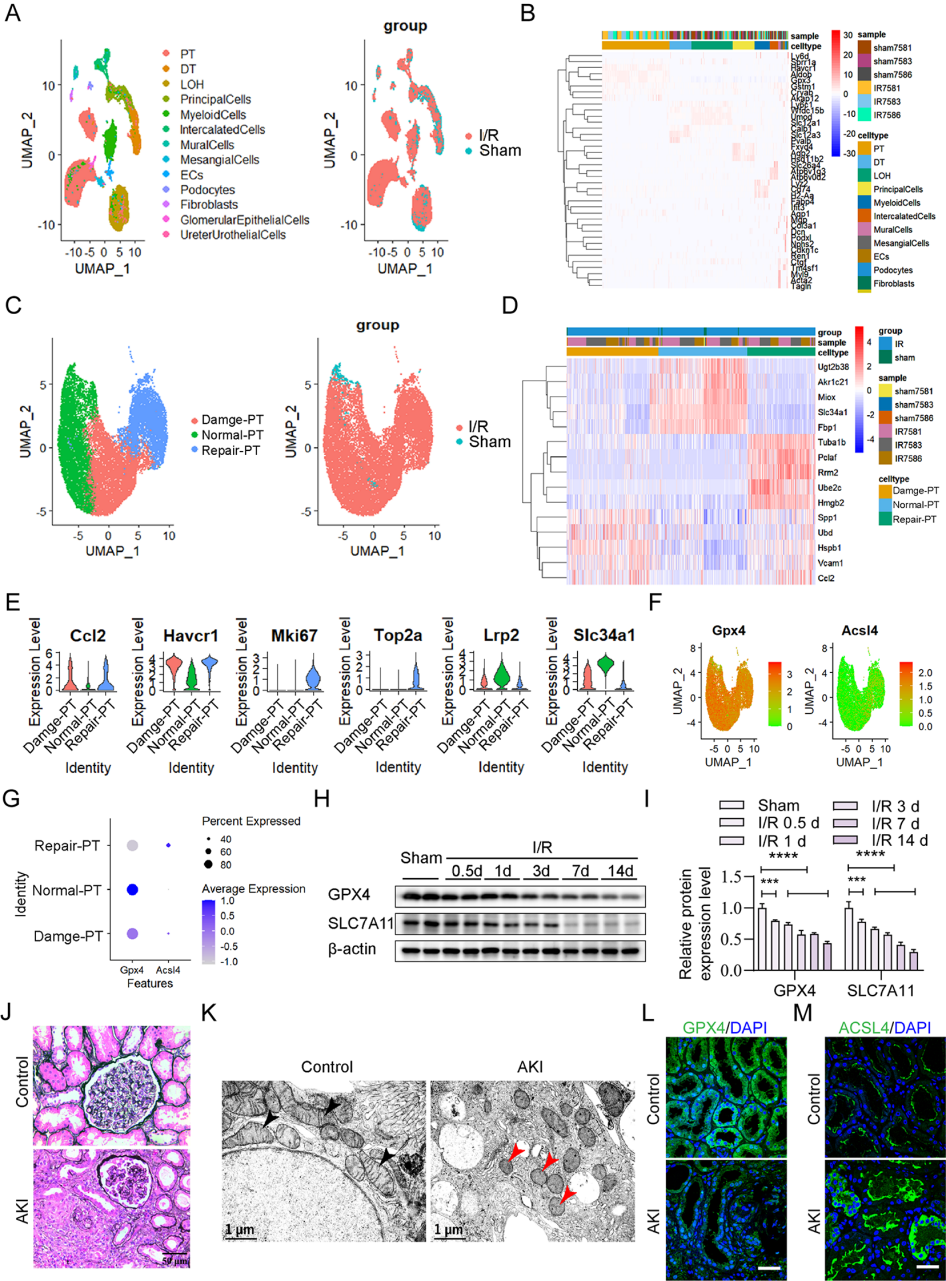
Renal tubular epithelial cells undergo ferroptosis in I/R-AKI. (**A**) Single-cell sequencing identified clusters in both sham-operated and I/R-operated kidneys at day 3 post-surgery, depicted in a *t-*distributed stochastic neighbor embedding (tSNE) map. PT, proximal tubule; DT, distal convoluted tubule; LOH, ascending loop of Henle; ECs, endothelial cells. (**B**) Average expression levels of mouse single cell type-specific genes are shown in pheatmap. Mean expression values of the genes were calculated in each cluster. The full list of cell types and genes is shown in Additional file1: Table S1. (C-E) Subtypes of PT with differentially expressed genes are illustrated in a tSNE plot (**C**), and the representative marker genes across the 3 subtypes of PT are depicted in pheatmap (**D**) and VlnPlot (E). (**F-G**) The expression levels of ferroptosis-associated genes *Gpx4* and *Acsl4* in the subtypes of PT were identified by FeaturePlot (**F**) and Dotplot (**G**). (**H**-I) Western blot (H) and statistical analysis (I) of the expression levels of GPX4 and SLC7A11 proteins in the renal cortex of sham-operated and I/R-operated mice post-surgery. ***p < 0.001, ****p < 0.0001. (**J-K**) TECs were visualized by periodic acid-schiff (PAS) staining (scale bar, 50 μm) (**J**) and transmission electron microscope (scale bars, 2 μm (left) and 1 μm (right), respectively) (**K**) in patients with minimal change disease (MCD) and AKI. Control, MCD. The black arrows indicate healthy mitochondria; the red arrows indicate damaged mitochondria. (**L-M**) Representative immunofluorescence staining of GPX4 (**L**) and ACSL4 (**M**) in AKI patients or controls. Scale bar, 40 μm

To better understand the role of ferroptosis in TECs, we performed single-cell sequencing analysis of kidney specimens from sham-operated and I/R-operated mice. Based on canonical markers derived from published studies and online databases [29], cell clusters were then delineated into thirteen distinct cell types (Fig. 1A, B). Given the pivotal role of renal TECs in I/R-AKI, we specifically focused on the proximal tubular epithelial cell cluster (PT) and further categorized it into three subtypes, including Damage-PT (identified by the marker genes *Ccl2* and *Havcr1*), Normal-PT (characterized by the marker genes *Lrp2* and *Slc34a1*) and Repair-PT (defined by the marker genes *Mki67* and *Top2a*) (Fig. 1C-E). Next, we analyzed the expression of genes involved in ferroptosis in these three TEC subtypes. As depicted in Fig. 1F **and G**, the anti-ferroptotic gene *Gpx4* was downregulated, while the ferroptotic gene *Acsl4* was upregulated in both Damage-PT and Repair-PT compared to that in Normal-PT. Further validation through western blot analysis demonstrated time-dependent downregulation of GPX4 and SLC7A11 proteins levels in the renal cortex of mice after I/R injury (Fig. 1H, I), indicating the induction of ferroptosis in TECs following I/R injury.

In addition to the animal models, renal biopsy specimens from 20 patients with acute tubular necrosis (ATN) and 20 patients without detectable ATN were enrolled in this study (Tables 1 and Fig. 1J). Transmission electron microscope corroborated the presence of shrunken mitochondria, a hallmark of ferroptosis, in patients with AKI (Fig. 1K). Using immunofluorescence, we found that GPX4 was decreased and ACSL4 was highly expressed in the renal tubules of patients with AKI, compared to those in the control group (Fig. 1L, M). These findings suggest that ferroptosis is induced in TECs of patients with AKI.

#### TECs are more sensitive to ferroptosis than apoptosis or necroptosis when suffering from H/R injury

We established an in vitro H/R model with the chemical hypoxia inducer CoCl_2_ for 24 h, followed by its removal. To investigate the impact of H/R on cell fate, HK-2 cells were exposed to CoCl_2_ at concentrations from 100 to 1000 µM for 24 h, followed by removal of CoCl_2_ for 3 h. MTT assay showed that H/R substantially reduced cell viability in a dose-dependent manner (Additional file 2: Fig. S1A). To elucidate the mechanism underlying cell damage and repair regulation during H/R, transcriptome sequencing was performed on both control and H/R-treated HK-2 cells. Gene set enrichment analysis (GSEA) demonstrated enrichment of the ferroptosis signaling pathway under H/R conditions (Additional file 2: Fig. S1B, C), suggesting the potential involvement of ferroptosis in the cellular response to H/R. Western blot analysis showed that GPX4 protein expression reached its lowest level 3 h after the removal of CoCl_2_, gradually recovering to normal level over the period of reoxygenation (Additional file 2: Fig. S1D and E). Additionally, GPX4 level was also decreased in HK-2 cells subjected to 24 h of hypoxia (1% O_2_) with varying periods of reoxygenation (21% O_2_) (Additional file 2: Fig. S1F and G). Together, these results indicate that H/R induces ferroptosis in HK-2 cells.

To better understand the role of ferroptosis and other forms of regulated cell death in HK-2 cells exposed to H/R, the levels of GPX4, p-MLKL, and PARP, commonly used markers of anti-ferroptotic, necroptotic and apoptotic processes, respectively, were measured by western blot analysis. As shown in Additional file 2: Fig. S1H-K, 3 h after the removal of CoCl_2_, low doses of CoCl_2_ induced a reduction in GPX4 protein expression without affecting the expression levels of p-MLKL or cleaved PARP in HK-2 cells, whereas high doses of CoCl_2_ promoted the expression of p-MLKL and cleaved PARP, and reduced the expression of GPX4 protein. Further observation showed that the decrease in cell viability caused by CoCl_2_ (200 µM)-induced H/R was markedly rescued by the ferroptosis inhibitors DFO and Fer-1, respectively, but not the apoptosis inhibitor Z-VAD-FMK or the necroptosis inhibitor Nec-1, whereas all of these inhibitors prevented the reduction in cell viability induced by CoCl_2_ (400 µM)-induced H/R (Additional file 2: Fig. S1L). Collectively, these data strongly suggest that HK-2 cells are more susceptible to H/R-induced ferroptosis than apoptosis or necroptosis, underscoring the predominant role of ferroptosis in cell death under H/R conditions.

#### SeNPs potently suppress ferroptosis in H/R-treated TECs

**Fig. 2:**
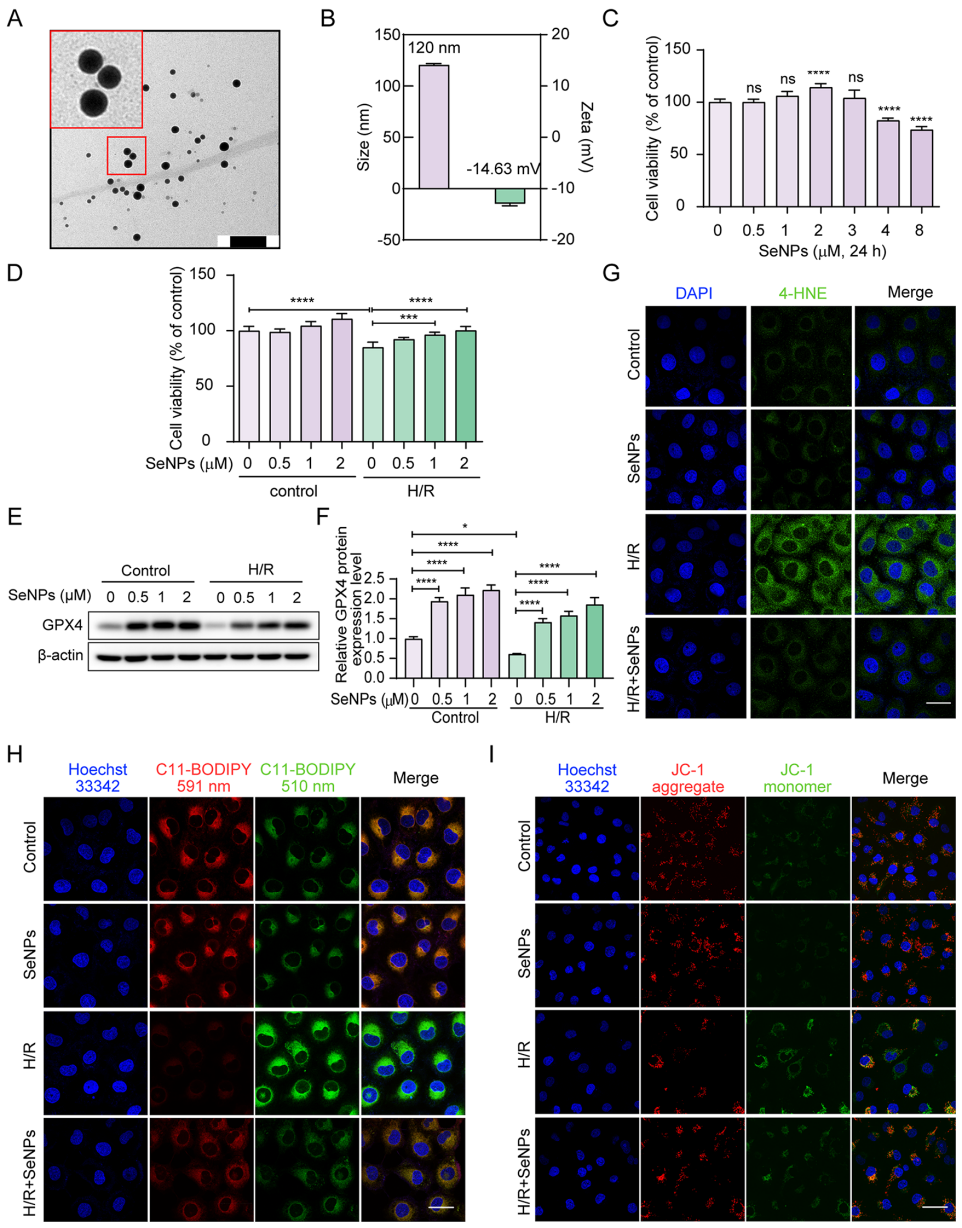
SeNPs potently suppress ferroptosis in H/R-induced HK-2 cells. (**A**) Representative transmission electron micrograph of SeNPs. Scale bar, 200 nm. (**B**) Hydrodynamic size distribution diagram and zeta potential of SeNPs. (**C**) HK-2 cells were incubated with the indicated doses of SeNPs for 24 h, and cell viability was measured with MTT assays. Each bar represents the mean of three determinations repeated in three separate experiments. ns, *p* > 0.05, ****p < 0.0001. (**D**) HK-2 cells were pretreated with or without 2 µM SeNPs for 1 h prior to 200 µM CoCl_2_ treatment, followed by a further 3 h incubation after CoCl_2_ was removed. MTT assay was used to measure cell viability. ***p < 0.001, ****p < 0.0001. (**E-F**) Western blot and statistical analysis of GPX4 protein expression in HK-2 cells in response to the indicated doses of SeNPs pretreatment for 1 h under CoCl_2_-induced H/R condition. (**G**) Lipid peroxidation was detected by immunofluorescence staining with an antibody against 4-HNE. Scale bar, 20 μm. (**H**) Intracellular lipid ROS levels by the staining of C11-BODIPY^581/591^ probe. Scale bar, 20 μm. (**I**) Changes in mitochondrial membrane potential by JC-1 staining. Scale bar, 50 μm

SeNPs were synthesized by the reaction of Na_2_SeO_3_ and vitamin C using lentinan as stabilizer through simple REDOX principle. It can be seen from the Transmission electron microscope image that SeNPs are basically evenly distributed and regularly round, with a diameter of 46 nm (Fig. 2A). The result of hydrodynamic size was 120 nm and the potential of SeNPs was − 14.63 mV (Fig. 2B), suggesting that they are conducive to the blood circulation of nanoparticles in the body.

Our previous studies confirmed the ability of SeNPs to inhibit apoptosis in various cells [18]. We initially explored the effects of SeNPs on HK-2 cells exposed to H/R. MTT assay revealed that 2 µM of SeNPs increased the viability of HK-2 cells under normal conditions (Fig. 2C), and remarkably ameliorated the reduction in cell viability induced by H/R (Fig. 2D). Prompted by the observation that H/R sensitized HK-2 cells to ferroptosis (Additional file 2: Fig. S1), we investigated the effects of SeNPs on ferroptosis in H/R-exposed HK-2 cells. Since GPX4 acts as one of the most pivotal enzymes resistant to lipid peroxidation and ferroptosis, we assessed its protein level to determine the efficacy of the antioxidant system. Western blot analysis revealed that SeNPs treatment caused a dose-dependent increase in GPX4 protein level in the presence or absence of H/R (Fig. 2E, F). Immunofluorescence staining was performed to verify the level of 4-HNE, a widely used biomarker of lipid peroxidation [30]. Figure 2G clearly showed the staining intensity of 4-HNE markedly increased in HK-2 cells in response to H/R treatment, which was reversed by SeNPs pretreatment. Lipid ROS accumulation, another hallmark of ferroptosis, was monitored using a C11-BODIPY^581/591^ probe [31]. As expected, SeNPs supplementation inhibited H/R-induced lipid ROS accumulation in HK-2 cells (Fig. 2H). Ferroptotic cells exhibite mitochondrial dysfunction, including the decreased mitochondrial membrane potential [32]. Using the JC-1 probe, we found that the mitochondrial membrane potential declined upon H/R treatment, indicating the loss of mitochondrial function, which could be markedly inhibited by SeNPs (Fig. 2I). Taken together, these data demonstrate that SeNPs potently suppress H/R-induced ferroptosis in TECs.

### SeNPs reduce ferroptosis in TECs and alleviate I/R-AKI in C57BL/6 mice

**Fig. 3:**
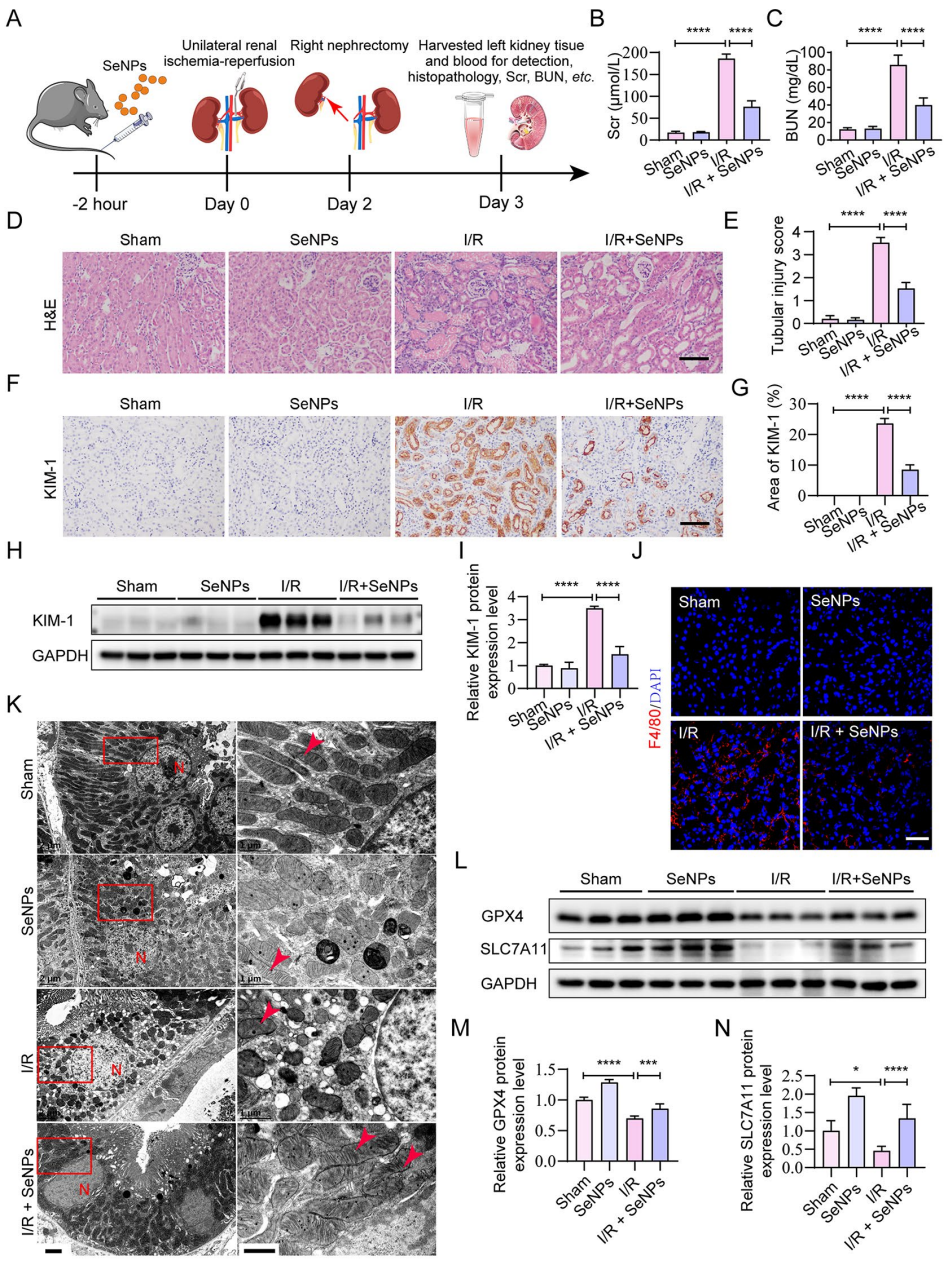
SeNPs alleviate I/R-induced kidney injury in C57BL/6 mice. (**A**) Schematic showing the experimental design for the unilateral I/R model of C57BL/6 mice intravenously injected with or without SeNPs (0.04 mg/kg). (**B-C**) Levels of Scr and BUN. (**D-E**) Kidneys from C57BL/6 mice 3 days after surgery were collected and subjected to H&E staining (**D**) and statistical analysis (**E**) to evaluate tubular injury score (**E**). Scale bar, 100 μm. (F-G) Representative images of the renal cortex stained with immunohistochemistry staining (**F**) and statistical analysis (**G**) of KIM-1 in kidney sections from Sham and I/R-operated mice, intravenously injected with or without SeNPs. Scale bar, 100 μm. (**H-I**) Western blot (**H**) and statistical analysis (**I**) of KIM-1 protein expression in the renal cortex. (**J**) Immunofluorescence staining of F4/80 in the renal cortex (scale bar, 50 μm). (**K**) Representative transmission electron micrographs of TECs in C57BL/6 mice (scale bar of the left column of the figure, 2 μm; scale bar of the right column of the figure, 1 μm). N indicates the nucleus; the arrow indicates mitochondria. (**L-N**) Western blot (**L**) and statistical analysis (**M-N**) of GPX4 and SLC7A11 protein expression in the renal cortex. *p < 0.05, ***p < 0.001, ****p < 0.0001

We further investigated the potential effect of SeNPs on I/R-AKI in vivo, following the experimental design outlined in Fig. 3A. When subjected to I/R injury, mice supplemented with SeNPs presented with lower levels of Scr and BUN (Fig. 3B, C). Kidney sections were evaluated for tubular injury by hematoxylin and eosin (H&E) staining and immunohistochemical staining with an anti-KIM-1 antibody. As depicted in Fig. 3D and E, I/R significantly damaged the kidneys, as evidenced by the loss of brush border, tubular dilatation, prominent cast formation and extensive inflammatory cell infiltration, all of which were remarkably mitigated by SeNPs treatment. Additionally, immunohistochemical staining for KIM-1, a marker of tubular injury, revealed a decrease in damaged tubules in renal I/R-treated mice following SeNPs supplementation (Fig. 3F, G). Western blot analysis corroborated these histological observations, showing a significant reduction in KIM-1 protein expression in SeNPs-treated I/R mice (Fig. 3H, I). Moreover, SeNPs treatment markedly inhibited the accumulation of F4/80 positive macrophages in the renal cortex of I/R mice (Fig. 3J), indicating a potential anti-inflammatory effect of SeNPs in I/R-AKI. Overall, these findings indicate that SeNPs attenuate I/R-AKI.

Reports have suggested that alterations in mitochondrial structure occur concurrently with ferroptosis, due to the close relationship between mitochondrial function and morphology [33]. Transmission electron microscope revealed that mouse TECs in the I/R group exhibited greater mitochondrial atrophy, increased mitochondrial membrane density, and reduced mitochondrial ridges compared to those in the sham group (Fig. 3K). Notably, these mitochondrial structural disorders were significantly ameliorated by SeNPs pretreatment. Furthermore, we observed a decrease in the expression levels of the anti-ferroptosis protein GPX4 and SLC7A11 in the renal cortex of I/R-treated mice compared to sham-treated mice, which was effectively reversed by SeNPs treatment (Fig. 3L-N). Taken together, our results indicate that the inhibitory effect of SeNPs on ferroptosis is responsible for the alleviation of I/R-AKI in C57BL/6 mice.

### SeNPs attenuate lysosomal iron accumulation and lysosomal dysfunction in H/R-induced TECs

**Fig. 4:**
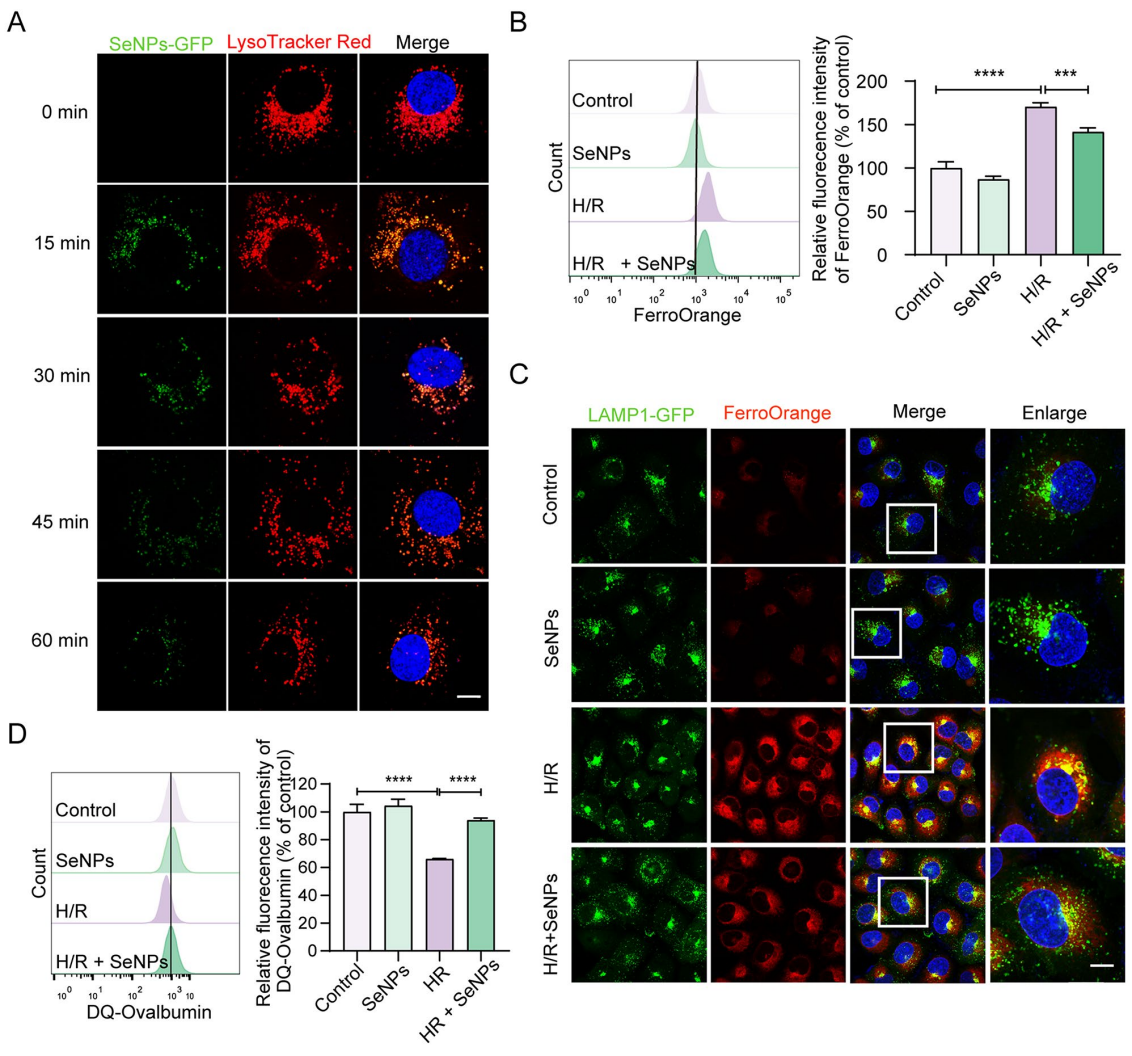
SeNPs suppress lysosomal iron accumulation and dysfunction induced by H/R in HK-2 cells. (**A**) Intracellular trafficking of SeNPs-GFP (2 µM) in HK-2 cells at the indicated time (scale bar, 10 μm). **(B**) HK-2 cells were pretreated, or not, for 1 h with 2 µM SeNPs followed by induction of hypoxia using 200 µM CoCl_2_ for 24 h and subsequent reoxygenation for 3 h. Intracellular Fe^2+^ levels were examined by flow cytometry using FerroOrange probes. (**C**) HK-2 cells transfected with LAMP1-mGFP plasmid were incubated for 1 h in the presence or absence of 2 µM SeNPs, and then incubated under CoCl_2_-induced H/R conditions. Lysosomal Fe^2+^ were detected using FerroOrange probe in LAMP1-mGFP-transfected cells under a fluorescence microscope (scale bar, 10 μm). The green puncta indicate lysosomes. (**D**) Flow cytometric analysis of DQ-ovalbumin staining in HK-2 cells with or without SeNPs pretreatment after exposure to CoCl_2_-induced H/R. ***p < 0.001, ****p < 0.0001. Each bar represents the mean of three determinations repeated in three separate experiments

Our previous study demonstrated the intracellular trafficking of SeNPs via endocytosis, which facilitates their internalization and signaling [34]. To investigate the intracellular dynamics of SeNPs, we employed a fluorescence imaging technique utilizing SeNPs-GFP and LysoTracker Red. As shown in Fig. 4A, SeNPs were initially internalized into the cytoplasm via endocytosis and were subsequently enriched within the lysosomes. The fluorescence intensity of SeNPs-GFP gradually diminished as they were quenched in the acidic lysosomal environment. Elevated lipid peroxidation may be triggered by excess Fe^2+^ levels [35]. Flow cytometry analysis revealed a significant reduction in the content of Fe^2+^ in H/R-induced HK-2 cells following SeNPs treatment via the detection of the Fe^2+^-specific probe FerroOrange (Fig. 4B). To further verify the localization of intracellular Fe^2+^ and its relation to lysosomes, master regulators of iron metabolism, we initially labeled LAMP1-mGFP (lysosome-associated membrane protein 1-mutated green fluorescent protein) plasmid-transfected HK-2 cells with FerroOrange. Our observations indicated that H/R-treated cells accumulated excess Fe^2+^, predominantly localized within lysosomes, which was markedly blocked by preincubation with SeNPs (Fig. 4C). Excess Fe^2+^ in lysosomes can seriously impair their function and structure via the Fenton reaction. As described in our previous study, DQ-ovalbumin, a self-quenched substrate for proteases, was used to assess the digestive capacity of lysosomes [36]. Flow cytometry analysis showed that SeNPs substantially alleviated the inhibitory effect of H/R on lysosomal digestion function in HK-2 cells (Fig. 4D). Collectively, these results indicated that H/R-induced lysosomal dysfunction may, to a certain extent, be a consequence of lysosomal iron accumulation and that SeNPs may exert their effects through the modulation of iron regulation.

### SeNPs suppress I/R-induced ferritinophagy in vivo and in vitro

**Fig. 5:**
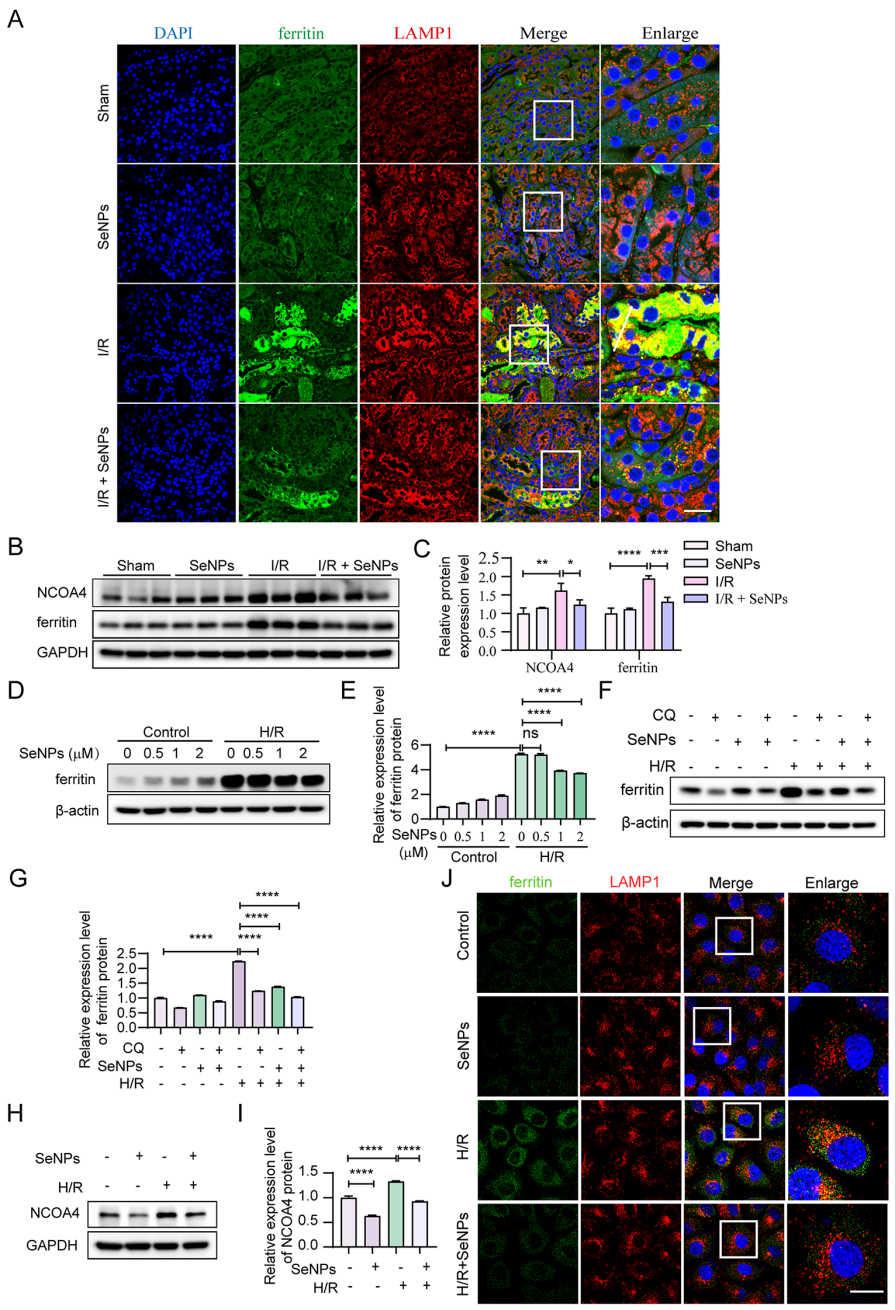
SeNPs suppress ferritinophagy in vivo and in vitro. (**A**) Immunofluorescence staining of LAMP1 and ferritin to detect ferritinophagy in the renal cortex of C57BL/6 mice subjected to either sham treatment or I/R injury, in the presence or absence of SeNPs administration (scale bar, 20 μm). (**B-C**) Western blot (**B**) and statistical analysis (**C**) of NCOA4 and ferritin protein expression in the renal cortex from sham-treated and I/R-treated C57BL/6 mice with or without SeNPs administration. (**D-E**) HK-2 cells were preincubated with or without the indicated doses of SeNPs for 1 h before the addition of 200 µM CoCl_2_ for 24 h, followed by the removal of CoCl_2_ for 3 h. The expression level of ferritin protein was then examined by western blotting. (**F-G**) Western blot (**F**) and statistical analysis (**G**) of ferritin protein level in HK-2 cells exposed to SeNPs (2 µM) or CQ (5 µM) for 1 h prior to CoCl_2_-induced H/R treatment or not. (**H-I**) HK-2 cells were incubated for 1 h in the presence or absence of 2 µM SeNPs, followed by incubation under CoCl_2_-induced H/R conditions. The cells were later subjected to western blot (**H**) and statistical analysis (I) of NCOA4 protein level and double immunofluorescencestaining of ferritin and LAMP1 (**J**). Fluorescence images show a representative experiment (scale bar, 20 μm)

Ferritinophagy, a selective type of autophagy, triggers ferroptosis by degrading ferritin within lysosomes, consequently releasing iron into labile iron pools and inducing iron overload [37]. Importantly, we found a marked increase in the colocalization of ferritin with LAMP1 in TECs in an in vivo I/R model, which was reversed by SeNPs treatment (Fig. 5A). To further elucidate the underlying mechanism by which SeNPs attenuate I/R-induced iron overload in TECs, we examined the protein levels of NCOA4, an identified ferritinophagy receptor [38], and ferritin. As shown in Fig. 5B **and C**, SeNPs significantly reduced the enhanced expression levels of NCOA4 and ferritin proteins in the renal cortex of C57BL/6 mice subjected to I/R injury.

We also confirmed the inhibitory effect of SeNPs on ferritinophagy in vitro. We examined the protein level of ferritin and found that, compared to H/R, SeNPs treatment reduced the ferritin protein level (Fig. 5D, E). Given that the level of ferritin protein decreases upon ferritinophagy [38], we utilized CQ, an autophagy inhibitor, to inhibit ferritinophagy and found that CQ decreased the level of ferritin upon H/R treatment alone or simultaneous treatment with SeNPs (Fig. 5F, G), providing preliminary evidence that SeNPs inhibited ferritinophagy under H/R conditions. Western blot analysis confirmed that SeNPs treatment significantly attenuated the enhanced expression of NCOA4 protein in HK-2 cells in the presence or absence of H/R (Fig. 5H, I). To directly visualize the effect of SeNPs on ferritinophagy, we performed immunofluorescence staining and found that SeNPs significantly alleviated the H/R-induced aggregation of ferritin and its colocalization with the lysosomes of HK-2 cells (Fig. 5J). Collectively, these findings indicate that SeNPs suppress H/R-induced ferritinophagy in TECs, which is consistent with the in vivo results.

## SeNPs inhibit ferroptosis partially via blocking XBP1/NCOA4-mediated ferritinophagy in TECs

**Fig. 6:**
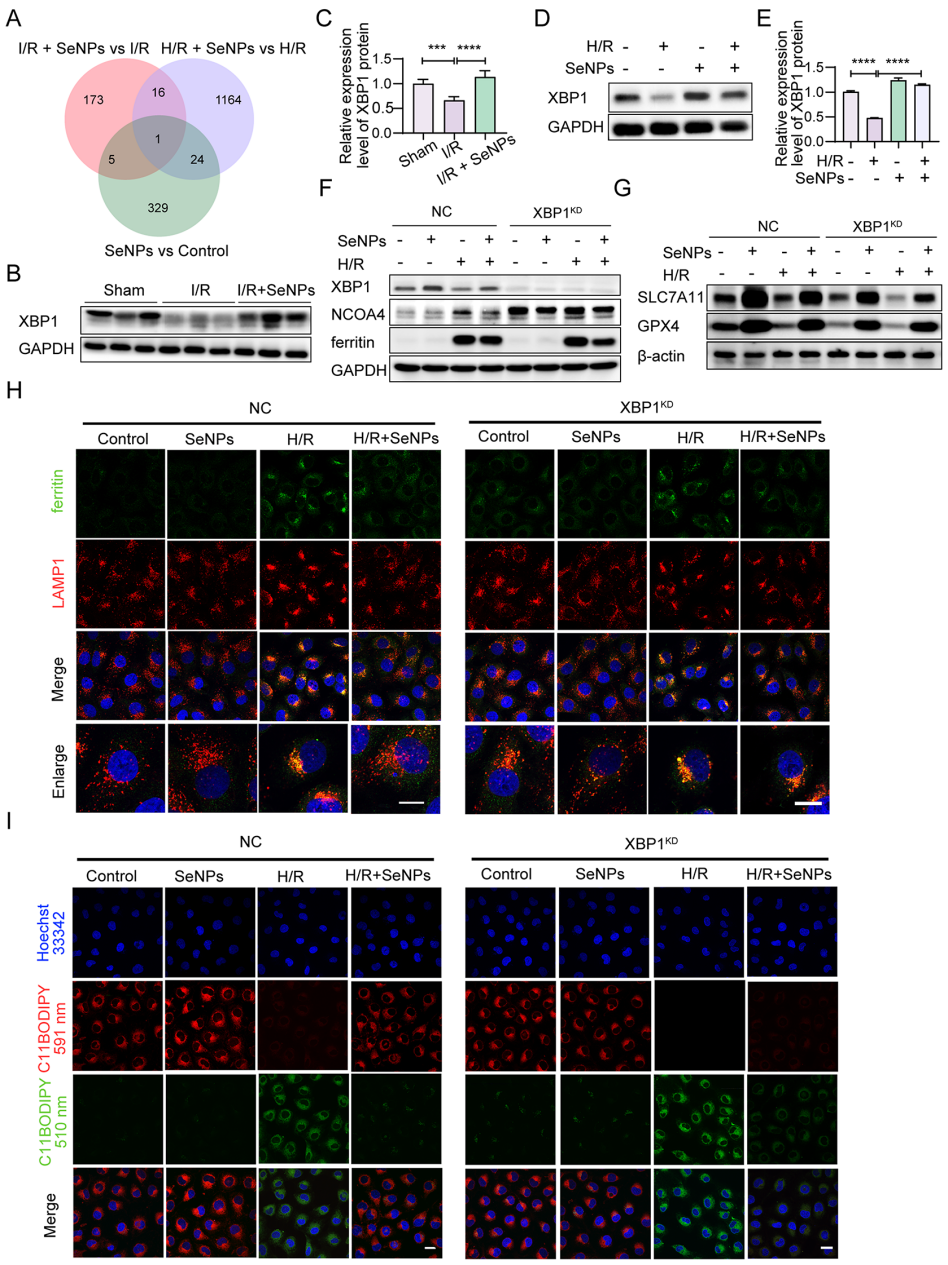
SeNPs partially inhibit ferroptosis via blocking XBP1/NCOA4-mediated ferritinophagy in TECs. (**A**) Venn diagram of differentially expressed and upregulated genes in the renal cortex of mice after 3 days of I/R operation and H/R-treated HK-2 cells, both of which were treated with or without SeNPs supplementation. (**B-C**) Western blot (**B**) and statistical analysis (**C**) of XBP1 protein in the renal cortex of mice subjected to either sham operation or I/R, supplemented with or without SeNPs. (**D-E**) Expression level of XBP1 protein in H/R-treated HK-2 cells treated with or without SeNPs was examined by western blotting. (**F**) Negative control (NC) and XBP1 knockdown (XBP1^KD^) HK-2 cells were cultured under control (normoxia) or H/R conditions in the presence or absence of SeNPs pretreatment for 1 h. Levels of XBP1 and ferritinophagic proteins NCOA4 and ferritin were assessed by western blotting. (**G**) Western blot analysis of anti-ferroptotic protein GPX4 and SLC7A11 levels. (**H**) Immunofluorescence staining of LAMP1 and ferritin to detect ferritinophagy in HK-2 cells (scale bar, 20 μm). (I) Detection of intracellular lipid ROS using C11-BODIPY^581/591^ probe (scale bar, 20 μm). Fluorescence images show a representative experiment. Statistical significance indicated as ***p < 0.001, ****p < 0.0001

To elucidate the potential mechanism by which SeNPs suppress ferritinophagy, transcriptome sequencing (RNA-seq) was performed to identify differentially expressed genes in the renal cortex of sham and I/R mice, as well as in HK-2 cells subjected to H/R, with or without SeNPs. The RNA-seq results showed that XBP1 was significantly upregulated in both the I/R in vivo model and the H/R in vitro model treated with SeNPs, as well as SeNPs treatment alone (Fig. 6A). Additionally, western blot analysis showed that I/R induced a decrease in XBP1 protein expression, which was markedly upregulated following SeNPs treatment (Fig. 6B, C), consistent with the results of H/R in vitro model (Fig. 6D, E). A further decrease in XBP1 expression level was observed after XBP1 knockdown in response to H/R treatment, which partially abolished the effect of SeNPs on XBP1, NCOA4 and ferritin proteins (Fig. 6F and Additional file 2: Fig. S2A-C). In addition, the co-localization analysis of dual immunofluorescence staining showed that exogenous downregulation of XBP1 expression eliminated the inhibitory effect of SeNPs on H/R-induced ferritin accumulation within lysosomes (Fig. 6H), further confirming that SeNPs play a role in the inhibition of ferritinophagy by upregulating XBP1 expression.

To further address whether SeNPs-inhibited ferroptosis was regulated through the upregulation of XBP1 expression, we first examined the protein levels of GPX4 and SLC7A11. As depicted in Fig. 6G and Additional file 2 Fig. S2 D-E, both GPX4 and SLC7A11 levels were significantly reduced in SeNPs-treated HK-2 cells after XBP1 knockdown compared to cells treated with SeNPs alone in the negative control (NC) group, in the presence or absence of H/R conditions. Lipid ROS were examined after the inhibition of XBP1 expression. Detection of lipid ROS using the C11-BODIPY fluorescent probe revealed that SeNPs decreased lipid ROS levels, whereas downregulation of XBP1 expression partially blocked this inhibitory effect (Fig. 6I). Taken together, these data demonstrate that SeNPs-induced upregulation of XBP1 inhibits ferroptosis via blocking ferritinophagy in HK-2 cells.

## Discussion

Although the pathogenesis of I/R-AKI has been partially clarified, including the production of free radicals, intracellular calcium overload, inflammatory effect and activation of the renin-vascular tensor-aldosterone system, effective prevention and treatment strategies remain elusive due to the lack of satisfactory results from in vitro and in vivo studies, along with the absence of clinical trials [39]. Studies have demonstrated the involvement of apoptosis, necrosis and autophagy in TECs in AKI [40, 41]. Our findings indicate that, TECs are more susceptible to ferroptosis in response to H/R, compared to apoptosis and necroptosis (Additional file 2: Fig. S1), suggesting that ferroptosis is a promising therapeutic target for treating TECs injury in I/R-AKI.

SeNPs, characterized by greater bioavailability and lower toxicity than selenium compounds, have emerged as promising immunomodulators and anticancer agents, due to their potent antioxidant and anticancer activities [42, 43]. Recent studies have indicated that SeNPs can attenuate I/R-AKI by promoting GPX1 expression and suppressing NLR family pyrin domain containing 3 (NLRP3) inflammasome activation [19]. However, the precise mechanism underlying the protective effects of SeNPs against I/R-AKI remains unclear. Given the evidence of the antioxidant ability of SeNPs, we investigated the potential role of SeNPs in regulating ferroptosis and preventing lipid peroxidation during I/R-AKI. Our findings revealed that SeNPs inhibited ferroptosis in TECs induced by H/R in vitro (Fig. 2) and I/R in vivo (Fig. 3). We previously reported that the disruption of lysosomal homeostasis, including lysosomal depletion and impairment of lysosomal biogenesis, contributes to tubular cell death and progression of kidney diseases [8, 44]. Further evidence suggests that the protective effects of SeNPs against ferroptosis are linked to the inhibition of lysosomal iron accumulation and the restoration of lysosomal homeostasis in TECs (Fig. 4C, D), implicating lysosomal dysfunction in ferroptosis in the context of I/R-AKI.

Previous studies have highlighted the detrimental effects of inflammatory cell infiltration, particularly by macrophages, on promoting cellular iron overload, exacerbating tissue injury and even fibrosis in heart, liver and kidney tissues under I/R conditions [45–47]. Consistent with these observations, our study confirmed that excessive macrophage infiltration in the kidneys (Fig. 3J) was accompanied by elevated level of ferritin protein co-localized with lysosomes (Fig. 5A) and accumulation of lysosomal iron in TECs under I/R conditions (Fig. 4C), all of which were inhibited by SeNPs supplementation.

Ferritin, a cytoplasmic iron-storage protein complex consisting of ferritin heavy chain 1 (FTH1) and ferritin light chain light (FTL) isoforms, undergoes autophagic degradation within lysosomes in response to iron deficiency, a process termed ferritinophagy, which maintains iron metabolism and homeostasis [48]. The level of ferritinophagy varies in cells of different tissue types and pathological states [49–52]. I/R injury is closely related to the excessive activation of ferritinophagy, which has been observed in various ischemia-related conditions, such as stroke models with I/R-induced neuronal injury and infarction models with I/R-induced myocardial injury [53, 54]. However, the specific role of ferritinophagy in I/R-AKI remains unclear. In the present study, we demonstrated that excess ferritin infiltrated TECs and accumulated within lysosomes during the initial phase of I/R, highlighting the importance of selectively inhibiting ferritinophagy to alleviate iron overload-induced TECs injury. In this study, we demonstrated that SeNPs effectively inhibited H/R-induced excessive activation of ferritinophagy (Fig. 5D-J) and prevented excessive accumulation of active iron within lysosomes (Fig. 4C).

XBP1, a key component of endoplasmic reticulum stress, plays a pivotal role in kidney diseases [55, 56]. Studies have shown that XBP1 downregulation shields kidneys from I/R injury by impeding HMG-CoA reductase degradation 1(HRD1)-mediated nuclear factor erythroid 2-related factor 2 (NRF2) ubiquitination [57]. However, the precise role of XBP1 in renal injury remains elusive. Nonetheless, XBP1 downregulation has also been reported to promote the transition from AKI to CKD [58]. In our study, we observed that TECs exposed to I/R i*n vivo* and H/R in vitro exhibited increased XBP1 protein level and low level of ferritinophagy following SeNPs pretreatment (Fig. 6A-E). Downregulation of XBP1 expression enhanced NCOA4 protein level and almost abolished the SeNPs-induced reduction in ferritinophagy (Fig. 6F, H), suggesting that SeNPs inhibit NCOA4-mediated ferritinophagy via XBP1 signaling in TECs. Further research is needed to elucidate the precise mechanism by which XBP1 regulates NCOA4-mediated ferritinophagy.

**Fig. 7:**
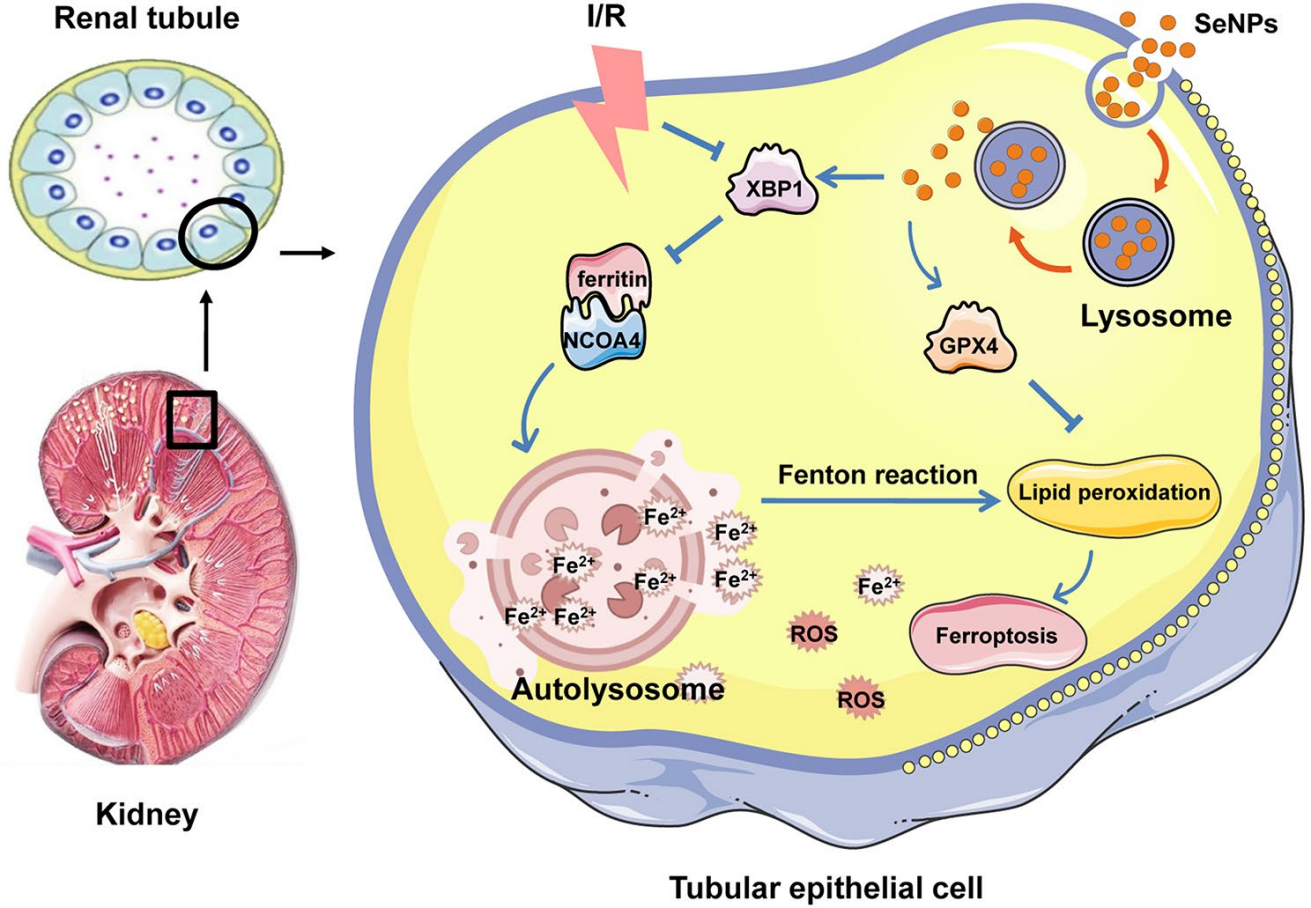
SeNPs attenuate I/R-induced ferroptosis of TECs by regulating XBP1/NCOA4-mediated ferritinophagy. SeNPs are initially internalized into the cytoplasm via endocytosis and subsequently enriched within the lysosomes in TECs. SeNPs then stimulate the expression level of XBP1 and block I/R-induced excessive activation of NCOA4-mediated ferritinophagy, which leads to decreased production of lysosomal Fe^2+^. In addition, SeNPs promote the expression of GPX4 in TECs. As a result, lipid peroxidation and ferroptosis are effectively attenuated

An intriguing finding of this work is the reduction in ferritin level after ferritinophagy inhibition induced by SeNPs. We cannot rule out the possibility that the decrease in ferritin level may be attributed to decreased ferritin synthesis. A previous study demonstrated that the transcription of ferritin could be stimulated by pro-inflammatory cytokines, such as interleukin (IL)-1β, IL-6 and tumor necrosis factor via the nuclear factor (NF)-κB pathway, while interferon gamma (IFNγ) and lipopolysaccharide (LPS) induced the degradation of iron regulatory protein 2 (IRP2) in a nitric oxide (NO)-dependent manner, leading to increased ferritin synthesis [59]. Notably, we and others have reported that SeNPs reduce the pro-inflammatory cytokines in various diseases [60, 61]. It is thus possible that SeNPs may play a role in the downregulation of ferritin synthesis.

Our findings also indicate that SeNPs-induced upregulation in the levels of GPX4 and SLC7A11 proteins was partially attenuated by XBP1 downregulation (Fig. 6G**)**. The SLC7A11/GPX4 axis is crucial in the regulation of ferroptosis [62], and the precise mechanism by which SeNPs influence this pathway requires further investigation.

## Conclusion

In summary, our present study identified a unique strategy for inhibiting ferroptosis by using SeNPs to disrupt XBP1/NCOA4-mediated ferritinophagy in TECs during I/R-AKI (Fig. 7). These findings shed light on a novel biological function of SeNPs, and elucidate the critical mechanism underlying their protective effects against I/R-AKI. We propose that blocking excessive activation of ferritinophagy could serve as a promising therapeutic target for I/R-AKI, and the application of SeNPs shows significant potential for future research and clinical intervention in kidney injury.

### Electronic supplementary material

Below is the link to the electronic supplementary material.

Supplementary Material 1

Supplementary Material 2

Supplementary Material 3

## Data Availability

No datasets were generated or analysed during the current study.

## Abbreviations

SeNPs: Selenium Nanoparticles
AKI: Acute Kidney Injury
CKD: Chronic Kidney Disease
ESRD: End-Stage Renal Disease
TECs: Tubular Epithelial Cells
I/R: Ischemia/Reperfusion
H/R: Hypoxia/Reoxygenation
NCOA4: Nuclear Receptor Coactivator 4
XBP1: X-box Binding Protein 1
LAMP1: Lysosomal-Associated Membrane Protein 1
GSEA: Gene Set Enrichment Analysis
GFR: Glomerular Filtration Rate
GPX4: Glutathione Peroxidase 4
CoCl_2_: Cbalt(II) Chloride Hexahydrate
p-MLKL: phospho-Mixed Lineage Kinase domain-Lke
PARP: cleaved Poly (ADP-Ribose) Polymerase
KIM-1: kidney Injury Molecule-1
SLC7A11: SoLute Carrier Family 7 member 11
Fer-1: Ferrostatin-1
Nec-1: Necrostatin-1
DFO: Deferoxamine
