# Supplementary material for: Selenium nanoparticles alleviate renal ischemia/reperfusion injury by inhibiting ferritinophagy via the XBP1/NCOA4 pathway
Source: Cell Commun Signal. 2024 Jul 25;22:376. doi: 10.1186/s12964-024-01751-2 (PMC11282718; doi:10.1186/s12964-024-01751-2)

**Additional file 2**

**Figure legend**

**Fig. S1 Renal TECs are more sensitive to H/R-induced ferroptosis than apoptosis or necroptosis.** (A) HK-2 cells were treated with the indicated dose of the hypoxia inducer CoCl_2_ for 24 h, followed by removal of CoCl_2_ for 3 h, and cell viability was measured with an MTT assay. The depiction “H 24 h/R 3 h” denotes hypoxia for 24 h followed by reoxygenation for 3 h. Statistical significance is indicated as ****p* < 0.001, *****p* < 0.0001. (B) Results of GSEA (KEGG pathways) for differentially expressed genes (DEGs) in HK-2 cells treated with H/R (200 μM CoCl_2_, H 24 h/R 3 h) compared to control (FDR and *p* < 0.05). (C) Enrichment plot displaying the ferroptosis pathway identified in the GSEA analysis. (D) HK-2 cells were incubated with or without 200 μM CoCl_2_ for 24 h and then reoxygenated by the withdrawal of CoCl_2_ for the indicated time, and the expression level of GPX4 was assessed by western blotting (D) and quantification analysis (E). (F-G) To induce H/R model *in vitro*, HK-2 cells were incubated in serum-free medium in an anaerobic chamber equilibrated with 1% O_2_, 5% CO_2_ and 94% N_2_ at 37°C for 24 h, and then subjected to normoxia conditions (95% air/5% CO_2_) for the indicated time periods. After cell lysis, the level of GPX4 was analyzed by western blotting (F) and quantification analysis (G). (H-K) HK-2 cells were treated with CoCl_2_ for 24 h at various doses and then incubated for an additional 3 h post-CoCl_2_ removal. The levels of the anti-ferroptotic protein GPX4, necroptotic protein p-MLKL and MLKL, and apoptotic protein PARP in these cells were assessed by western blotting and quantification analysis. (L) HK-2 cells were pretreated for 1 h in the presence or absence of inhibitors against ferroptosis (100 μM DFO and 2 μM Fer-1), necroptosis (40 μM Nec-1) and apoptosis (20 μM Z-VAD-fmk) respectively, and then exposed to CoCl_2_ for 24 h followed by the withdrawal of CoCl_2_. The cells were collected 3 h later and subjected to MTT assay. ns means *p* > 0.05, **p* < 0.05, ***p* < 0.01, ****p* < 0.001, *****p* < 0.0001.

**Fig. S2** **Quantification analysis of protein levels examined by western blotting.** (A-E) Statistical analysis of the levels of XBP1, NCOA4 and ferritin proteins (detected by western blotting and shown in **Fig. 6F**) (A-C), SLC7A11 and GPX4 proteins (detected by western blotting and shown in **Fig. 6G**) as well (D-E). ns, *p* > 0.05, ***p* < 0.01, ****p* < 0.001, *****p* < 0.0001.

**Figure**

**Fig. S1**


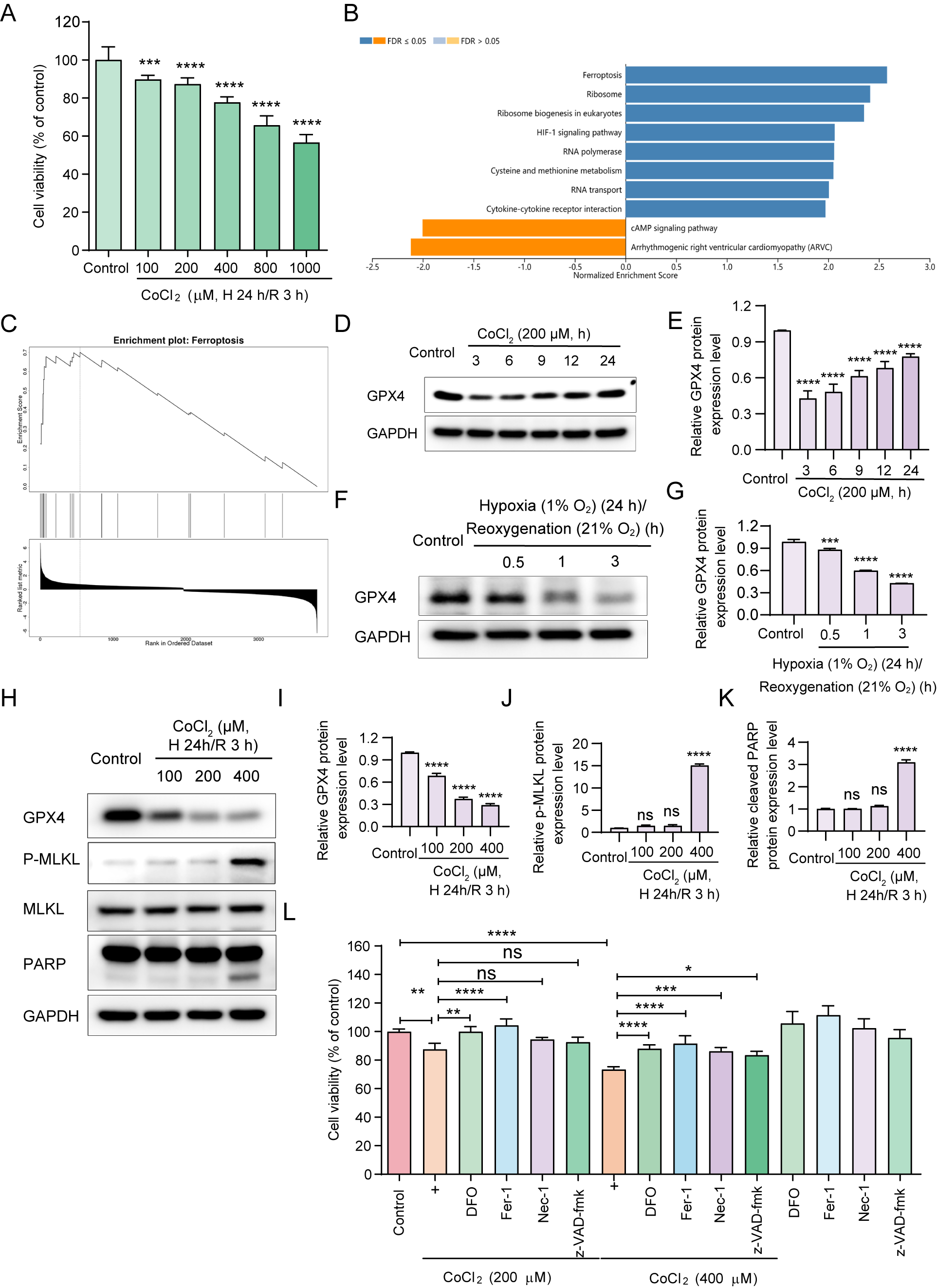


**Fig. S2**


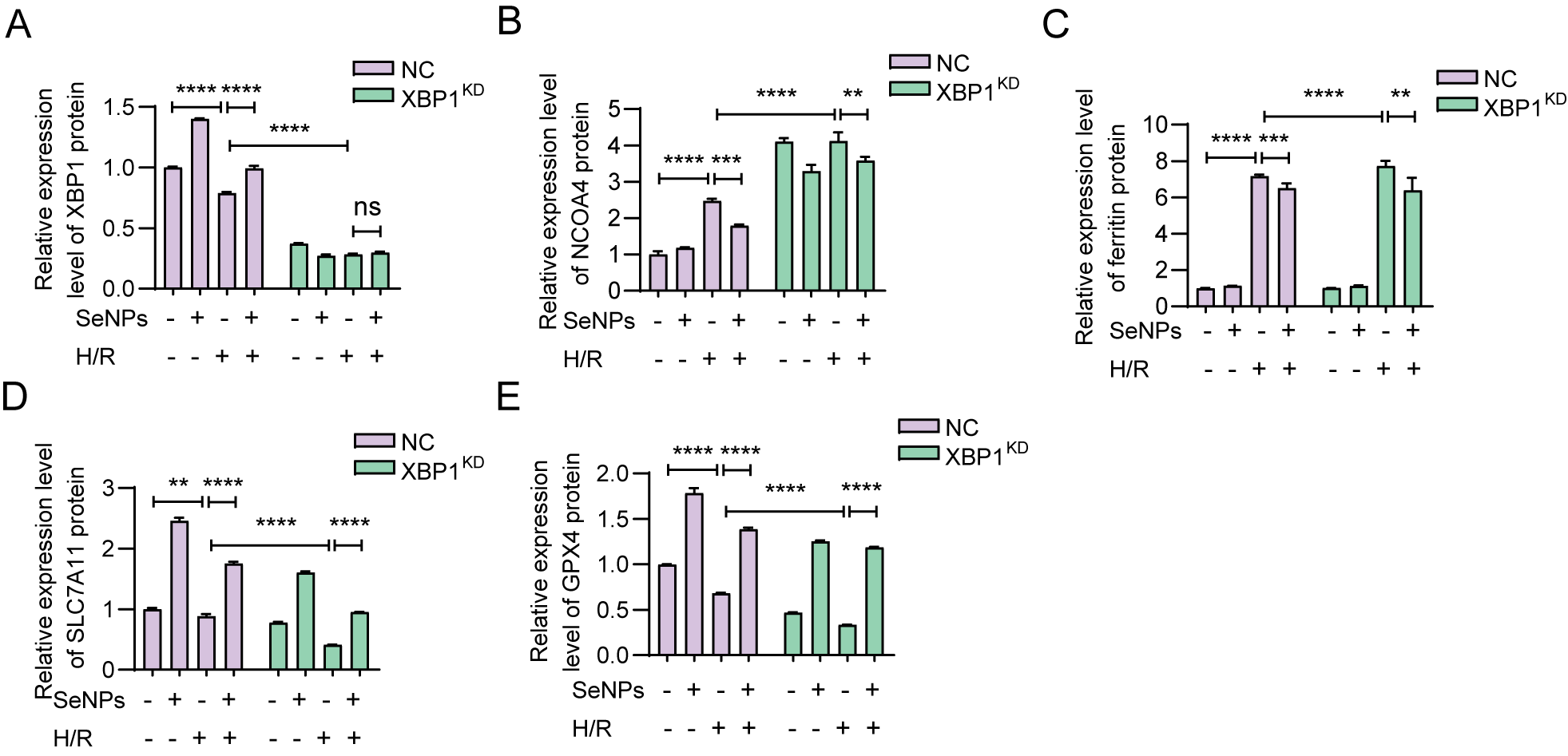

Supplement: Supplementary file 1 — Supplementary Material 1 [file 12964_2024_1751_MOESM1_ESM.docx]
