# Supplementary figures and images for: Selenium nanoparticles alleviate renal ischemia/reperfusion injury by inhibiting ferritinophagy via the XBP1/NCOA4 pathway
Source: Cell Commun Signal. 2024 Jul 25;22:376. doi: 10.1186/s12964-024-01751-2 (PMC11282718; doi:10.1186/s12964-024-01751-2)

**Additional file 3**

**Figure: raw image of western blots**

**Fig. S3**


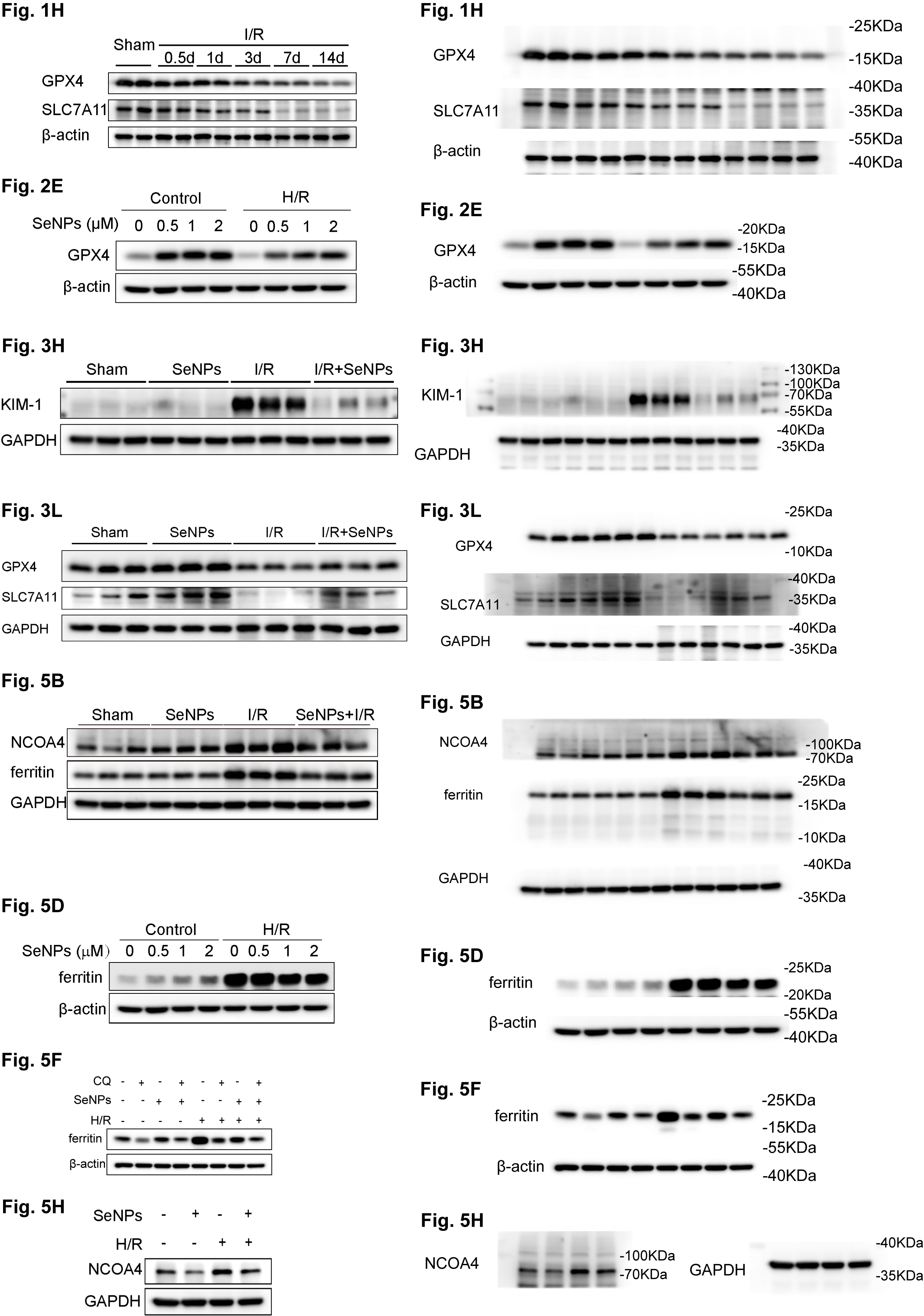


**Fig. S4**


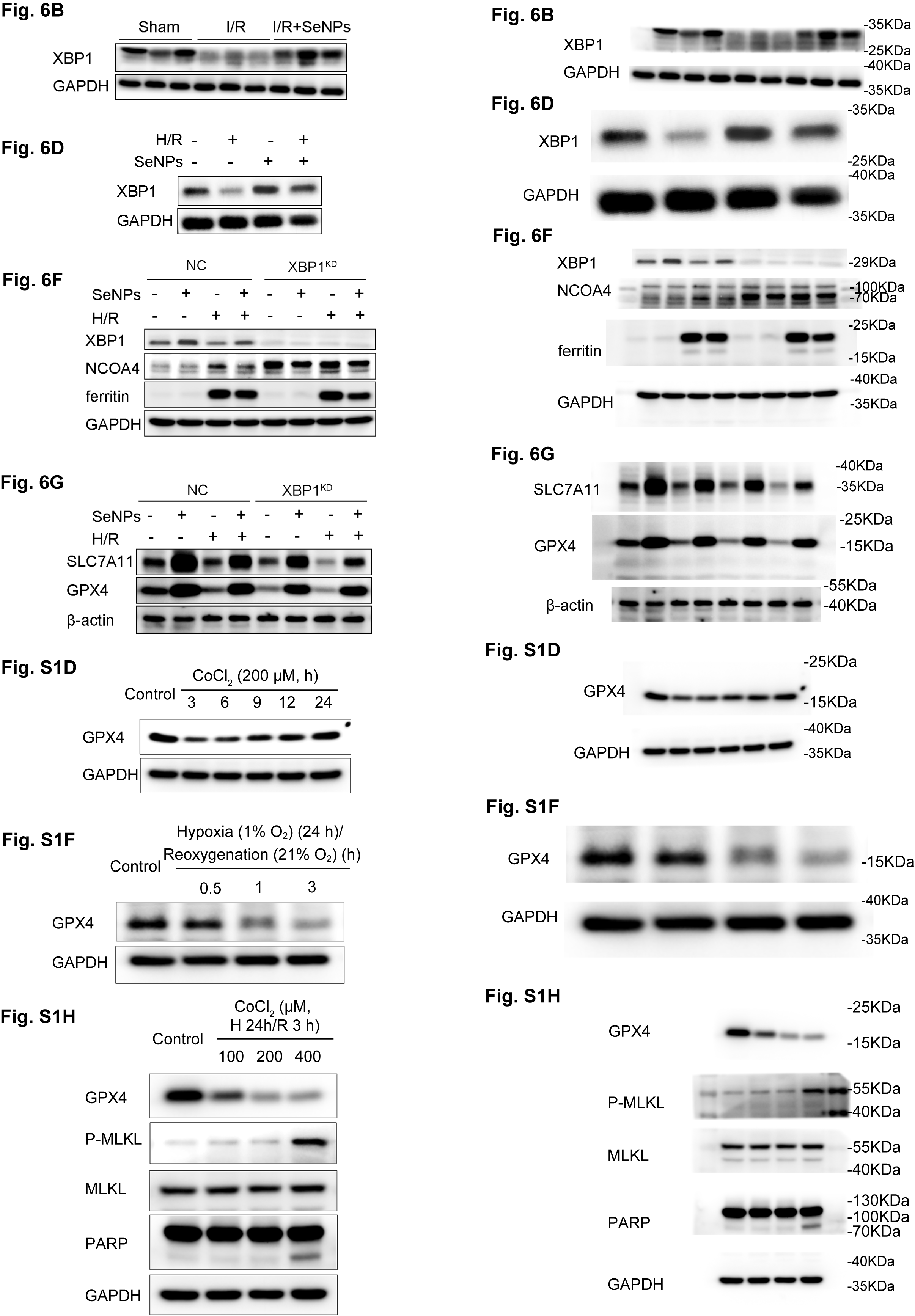

Supplement: Supplementary file 2 — Supplementary Material 2 [file 12964_2024_1751_MOESM2_ESM.docx]

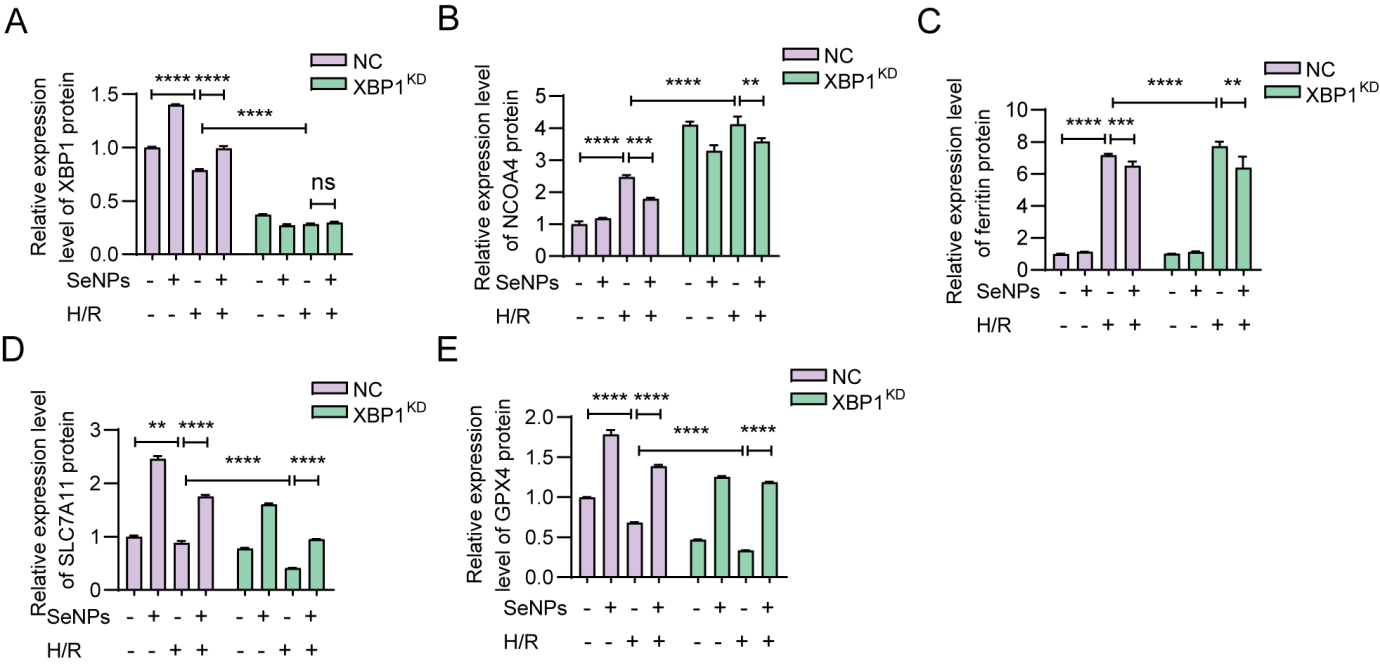


Fig S1


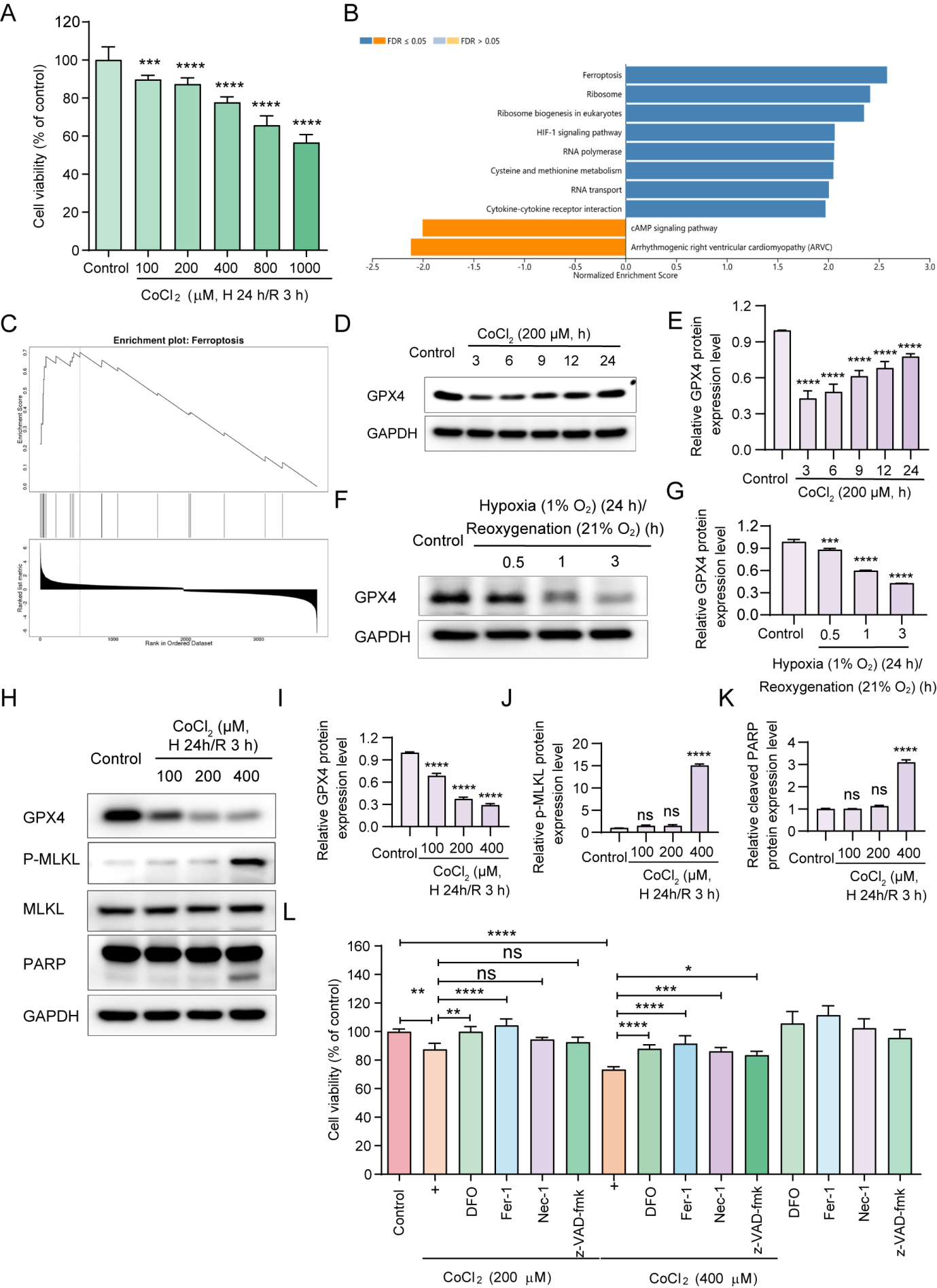


Fig S2

Supplement: Supplementary file 3 — Supplementary Material 3 [file 12964_2024_1751_MOESM3_ESM.docx]
